# Effect of sleep deprivation and NREM sleep stage on physiological brain pulsations

**DOI:** 10.3389/fnins.2023.1275184

**Published:** 2023-12-01

**Authors:** Heta Helakari, Matti Järvelä, Tommi Väyrynen, Johanna Tuunanen, Johanna Piispala, Mika Kallio, Seyed Mohsen Ebrahimi, Valter Poltojainen, Janne Kananen, Ahmed Elabasy, Niko Huotari, Lauri Raitamaa, Timo Tuovinen, Vesa Korhonen, Maiken Nedergaard, Vesa Kiviniemi

**Affiliations:** ^1^Oulu Functional Neuroimaging (OFNI), Department of Diagnostic Radiology, Oulu University Hospital, Oulu, Finland; ^2^Research Unit of Health Sciences and Technology, Faculty of Medicine, University of Oulu, Oulu, Finland; ^3^Clinical Neurophysiology, Oulu University Hospital, Oulu, Finland; ^4^Center of Translational Neuromedicine, University of Copenhagen, Copenhagen, Denmark; ^5^Center of Translational Neuromedicine, University of Rochester, Rochester, NY, United States

**Keywords:** fluid transport, glymphatic, homeostasis, non-REM sleep, sleep pressure

## Abstract

**Introduction:**

Sleep increases brain fluid transport and the power of pulsations driving the fluids. We investigated how sleep deprivation or electrophysiologically different stages of non-rapid-eye-movement (NREM) sleep affect the human brain pulsations.

**Methods:**

Fast functional magnetic resonance imaging (fMRI) was performed in healthy subjects (*n* = 23) with synchronous electroencephalography (EEG), that was used to verify arousal states (awake, N1 and N2 sleep). Cardiorespiratory rates were verified with physiological monitoring. Spectral power analysis assessed the strength, and spectral entropy assessed the stability of the pulsations.

**Results:**

In N1 sleep, the power of vasomotor (VLF < 0.1 Hz), but not cardiorespiratory pulsations, intensified after sleep deprived vs. non-sleep deprived subjects. The power of all three pulsations increased as a function of arousal state (N2 > N1 > awake) encompassing brain tissue in both sleep stages, but extra-axial CSF spaces only in N2 sleep. Spectral entropy of full band and respiratory pulsations decreased most in N2 sleep stage, while cardiac spectral entropy increased in ventricles.

**Discussion:**

In summary, the sleep deprivation and sleep depth, both increase the power and harmonize the spectral content of human brain pulsations.

## Introduction

Four decades ago, Borbely presented a sleep model that combined the homeostatic sleep pressure concept and circadian rhythm (Borbély, 1982; Borbély et al., 2016). To date, the physiological mechanisms of sleep pressure are not fully established. However, it is known that extrusive sleep pressure during subsequent recovery sleep increases the amplitude and amount of delta waves (0.5–4 Hz) (Marzano et al., 2010; Hubbard et al., 2020). Several studies link the increase in delta waves during sleep with accelerated cerebrospinal fluid (CSF) flow and brain clearance (Xie et al., 2013; Fultz et al., 2019; Helakari et al., 2022), suggesting that sleep pressure might boost homeostatic cellular processes in a manner extending beyond the well-recognized changes in neural activity patterns.

In our previous study, we presented how three pulsation mechanisms, namely vasomotor, respiratory, and cardiovascular pulsations, intensified during non-rapid-eye-movement (NREM) sleep in the same human brain regions showing increased delta power (Helakari et al., 2022). Cardiovascular (Iliff et al., 2013; Mestre et al., 2018), respiratory (Dreha-Kulaczewski et al., 2015; Vinje et al., 2019) and vasomotor (Kiviniemi et al., 2016; van Veluw et al., 2020) pulsations have all previously been suggested to drive CSF flow or metabolic solute transport (Rasmussen et al., 2018). In contrast to accelerated brain clearance, decreased slow wave activity and sleep efficiency likely propagates to β-amyloid accumulation over the years (Winer et al., 2020). Molecular imaging research shows that acute sleep deprivation increases β-amyloid levels with duration of sleep deprivation (Kang et al., 2009; Shokri-Kojori et al., 2018), and that the subsequent recovery sleep does not fully rescue impaired solute clearance (Eide et al., 2022). The converse relationship seems to hold, as β-amyloid expression induces fragmented and reduced sleep (Tabuchi et al., 2015). While the investigation of sleep pressure and recovery sleep offers a window to understanding the homeostatic need for sleep, measuring physiological changes across different sleep stages should increase our understanding of the physiological mechanisms that occur during sleep.

Awake state in EEG recordings is characterized by dynamic changes from fast gamma and beta activity to slower alpha and theta waves. NREM sleep is divided into stages extending from N1 drowsiness to N3 deep sleep (American Academy of Sleep Medicine, 2017). The N1 stage represents a transition from wakefulness to sleep; it includes increased amounts of theta activity (4–8 Hz) and vertex waves, and the stage varies between arousal episodes and sleep. The N2 stage is considered as intermediate sleep, characterized by EEG as manifesting K-complexes and sleep spindles (12–16 Hz) that support memory consolidation. Additionally, N2 sleep is characterized by larger amplitude physiological changes such as decreasing heart rate, blood pressure, and body temperature, and a greater regulation of respiration (Somers et al., 1993; Cabiddu et al., 2012; Malik et al., 2012). N3 stage sleep is considered the deepest stage of sleep. In the (AASM criteria American Academy of Sleep Medicine, 2017), it is characterized to include 20% of slow wave activity (0.5–2 Hz) sleep (delta waves). In a normal sleep cycle, N1 occurs first followed by N2 and N3 that alternates with rapid-eye-movement (REM) sleep so that more N3 occurs early during sleep and the amount of REM sleep increases as sleep progresses. In addition to EEG, fMRI serves as a potential tool to study physiological changes across different brain states. Importantly, increased very low-frequency (VLF) fluctuations measured with fMRI occur after sleep deprivation (Gao et al., 2015) and during lowered vigilance states and sleep (Fukunaga et al., 2006; Horovitz et al., 2008; Chang et al., 2016; Liu et al., 2018), which are global and of particular prominence in the visual and auditory cortices. Recent article presented how falling asleep sleep can be locally characterized from fMRI (Song et al., 2022). Importantly, their data indicated that in fMRI, the low frequencies (<0.1 Hz) were prominent in light sleep N1-2 and higher frequencies (0.1–0.2 Hz) in deep N3 sleep.

While conventional fMRI can reveal hemodynamic changes in VLF, faster techniques allow the recording of pulsation changes in the brain in the frequency range of respiration and heart rate (Klose et al., 2000; Posse et al., 2013; Yamada et al., 2013; Dreha-Kulaczewski et al., 2015; Sloots et al., 2020). The fast fMRI method known as MREG, with its 10 Hz sampling rate, serves as a new tool to study the three classes of brain pulsations, separately and without overlap or aliasing (Zahneisen et al., 2012; Assländer et al., 2013; Kiviniemi et al., 2016). We have earlier shown that physiological monitoring signals, respiratory belt or end-tidal carbon dioxide (EtCO_2_) and fingertip peripheral (SpO_2_) strongly correlate with MREG (*r* > 0.92) (Tuovinen et al., 2020; Järvelä et al., 2022; Kananen et al., 2022). Therefore, separating these phenomena in MREG makes it possible to study physiological effects in the brain, and their changes across arousals states.

In this study, we tested the hypotheses that pulsation stability and strength are highly dependent on brain arousal state, specifically focusing on the condition of wakefulness, the effects of sleep deprivation, and across NREM sleep stages with differential EEG signatures. We used spectral power analysis to measure intensity and spectral entropy of the signals as an index of spectral stability of the pulsations as in our previous study (Helakari et al., 2022). We also measured signals in the segmented 4th ventricle, CSF, gray matter (GM), and white matter (WM) to investigate whether pulsations differ depending on the brain tissue. We propose that sleep pressure due to acute deprivation and changes in sleep stages strongly affect the brain physiology, which would support and extend the original Borbely model of a restorative function of sleep to include a homeostatic mechanism, namely brain fluid clearance.

## Materials and methods

### Subjects and study design

The study was approved by the Regional Ethics Committee of the Northern Ostrobothnia Hospital District. Written informed consent was obtained from all subjects, according to requirements of the Declaration of Helsinki. Subjects were recruited from local university student and hospital personnel e-mail lists. All subjects were healthy as assessed by an interview and met the following inclusion criteria: no neurological or cardio-respiratory diseases, no continuous medication, non-smokers and no possibility of pregnancy.

We included two data sets in the analysis (Supplementary Table 1). The first (1) dataset included 13 subjects (aged 25.9 ± 4.2 years, 5 females) from the study group in Helakari et al. (2022) and the second (2) dataset ten subjects (28.4 ± 5.7 years, 7 females), 23 subjects in total (27.0 ± 4.9 years, 12 females). There were no overlap in the subjects between groups (1) and (2). Due to MRI scanner availability, the subjects in dataset (1) were scanned awake in the afternoon (4–6 PM) while awake eyes open after a normal night’s sleep and while asleep after sleep deprivation (6–8 AM), as previously described (Helakari et al., 2022). We instructed the subjects not to consume caffeine for at least 4 h preceding the Awake scan session and 8 h preceding the Sleep scan session. Also, we instructed them not to consume any alcohol during the sleep deprivation. Subjects were allowed to be home during sleep deprivation night, but to ensure sleep deprivation, smart ring data was measured. We instructed subjects to use the smart ring at least 24 h preceding the both scans. Smart ring has been especially designed to measure sleep, and it records photoplethysmogram (250 Hz), upper limb motion (3-D accelerometer, 50 Hz) and skin temperature (1/min) to discover this information. Validations studies suggest that smart ring performs well to separate wakefulness from sleep (de Zambotti et al., 2017; Mehrabadi et al., 2020).

Subjects in dataset (2) were scanned awake eyes open in the afternoon (4–6 PM) after a normal night’s sleep, and while asleep in the evening during regular sleep time (10 PM – 12 AM). Awake and sleep scans for group (1) were performed during the same week as presented in Helakari et al. (2022). For group (2), awake and sleep scans were performed within two months, depending on available scanning schedules.

To study differences between sleep deprived and non-sleep deprived sleep, we compared 12 sleep deprived to 8 non-sleep deprived subjects. For the sleep stage analysis we had the following amounts of subjects N1 (12 sleep deprived, 1 subject with continuous N1 stage from eyes closed scan from sleep deprivation population and 8 non-sleep deprived evening scans) and for N2 (10 sleep deprived and 4 non-sleep deprived evening scans) (Supplementary Table 1).

As N1 and N2 sleep are known to differ in characteristics seen in EEG (American Academy of Sleep Medicine, 2017), we wanted to study these two stages separately. We chose subjects in whom the MREG data included two-minute epochs of continuous N2 state, and similarly two-minute epochs of N1 and awake states, which enabled an accurate comparison between sleep stages. Therefore, we used four subsequent 30 s epochs as continuous two-minute signal for awake, N1 and N2. We chose the two-minute data in any location in 10-20-min data, that had sufficient EEG for sleep scoring and included continuous sleep stage. For the analysis, we took all the continuous two-minute data that were available in each stage, and due to lack of, e.g., N2 stage in non-sleep deprived subjects, the amount of subjects was four. As MREG is a fast sequence, sampled with 10 Hz whole brain sampling rate, the two-minute analysis brings a statistically relevant information of the signal with 1,200 brain volumes.

### Data acquisition

All subjects were scanned in Oulu (Finland) using a Siemens MAGNETOM Skyra 3T (Siemens Healthineers AG, Erlangen, Germany) scanner with a 32-channel head coil. The MREG, fast fMRI sequence (Zahneisen et al., 2012; Assländer et al., 2013; Lee et al., 2013; Hennig et al., 2021) was used in synchrony with a previously described multimodal scanning setup (Korhonen et al., 2014). The following parameters were used for MREG: repetition time (TR = 100 ms), echo time (TE = 36 ms), and field of view (FOV = 192 mm), 3 mm cubic voxels and flip angle (FA = 5°). In MREG, SAR value is only 2% (Jacobs et al., 2014) of conventional EPI that already has low SAR value. There are two main reason for that: (1) low flip angle (in our study only 5°) and (2) longer excitation time (100 ms 3 D vs. 62 ms 2 D).

Importantly, the interscan crusher gradient is set to 0.1 to optimize sensitivity for physiological signal while preventing still the development of stimulated echoes and slow signal drifts. L2-Tikhonov regularization with lambda 0.1 were used for reconstruction, with the latter regularization parameter determined by the L-curve method with a MATLAB recon-tool provided by the sequence developers (Hugger et al., 2011). Dynamic off-resonance correction in k-space (DORK) was used to reduce respiratory motion and B0-field artifacts in the datasets. Parameters for three-dimensional structural T1 MPRAGE were TR = 1900 ms, TE = 2.49 ms, FA = 9°, FOV = 240 mm, and slice thickness 0.9 mm.

EEG was recorded using the MRI compatible Electrical Geodesics (EGI, Magstim Company Ltd., Whitland, UK) MR-compatible GES 400 system, with a 256-channel high density net and previously described settings (Helakari et al., 2022). We took special care to avoid loops of EEG wires. Respiratory belt and SpO_2_ and anesthesia monitor data (ECG, SpO_2_ and EtCO_2_, Datex-Ohmeda S/5 Collect software) were measured in synchrony with the EEG, as described previously (Korhonen et al., 2014).

### Preprocessing and analysis of MREG data

Preprocessing of MREG data was obtained with FSL 5.09 BET software (Smith, 2002; Jenkinson et al., 2012), and framewise head motion correction was done with FSL 5.08 MCFLIRT software (Jenkinson et al., 2012) MCFLIRT relative or absolute mean displacement values (mm) of scans (Awake versus Sleep scan 1 or Sleep scan 2 scan in dataset 1 and Awake versus Sleep scan in dataset 2) did not differ significantly (*p*_all_ > 0.094). The highest spikes in the MREG data time series were removed using the 3dDespike function in AFNI (Analysis of Functional NeuroImages, v2) (Cox, 1996), which was deemed a reasonable method to diminish the effects of motion. After standard preprocessing and despiking, the data were registered to the Montreal Neurological Institute (MNI152) space at 3-mm resolution, for comparable analysis between subjects.

We chose to use two-minute data windows (1,200 time points), this being the maximum length that provided continuous N2, N1, and awake segments from most of the subjects. In the sleep deprived subjects (1), the data were chosen from 10-min of awake eyes open data and 20-min of sleep data. In the non-sleep deprived subjects (2), awake data were chosen similarly as in (1), and sleep data from 10-min epochs of MREG data. In our previous article, we showed that 32% of eyes closed epochs were scored as sleep, and thus we used eyes open data to confirm wakefulness. Also previous literature supports the choice to use eyes open data if wanting to ensure the wakefulness (Tagliazucchi and Laufs, 2014). To include also VLF in the analysis, we filtered VLF for 0.01–0.08 Hz. The minimum value was chosen according to the length of the data string and maximal value according to the respiratory frequency range, that in some subjects extended to 0.08 Hz (meeting the noise level criteria, by visual inspection). A full band minimum value was similarly chosen for spectral entropy analysis (frequency range 0.01–5 Hz).

### Frequency ranges for pulsation analysis were determined from physiological EtCO2 and SpO2 signals

Individual frequency ranges for respiratory and cardiac frequency bands were determined mainly from 10-min EtCO_2_ and SpO_2_ signal data measured with Datex-Ohmeda S/5 Collect Monitor in synchrony with MREG. In case of incomplete 10-min data, we used 5-min recordings measured with the Skyra physiological monitoring system, which gave up to 5-min recordings in six cases for the respiratory waves and three cases for cardiac, and for global MREG in two cases both for respiratory and cardiac pulsations. Correlation analysis between physiological monitoring (respiratory belt or EtCO_2_ and SpO_2_) vs. MREG indicates very high correlation r > 0.92 (Tuovinen et al., 2020; Järvelä et al., 2022; Kananen et al., 2022). Therefore, changes in arousals states such as slow variability of cardiorespiratory physiology are also directly reflected in MREG signal of the brain. With the use of individual frequency ranges, we wanted to focus specifically on the phenomena under consideration (Järvelä et al., 2022), therefore excluding noise, harmonics and heterodynes. Harmonics arising from the principal frequency of the event, e.g., respiration/cardiac are observed in multiples of this frequency across the power spectrum. Heterodynes are the modulation imposed by lower frequencies upon higher frequency events and their respective principal frequencies. The criteria for choosing the frequency range entailed locating the minimum point, when the spectrum increased above noise level and the maximum point when the spectrum decreased back to the noise level. The respiratory frequency range varied between 0.08 and 0.49 Hz and cardiac frequency between 0.51 and 1.56 Hz in datasets (1) and (2). When determining the frequency ranges (from minimum to maximum), we also obtained the peaking value to determine the respiratory and heart rates of the subjects during each ten-minute epoch.

### Preprocessing and analysis of EEG data

EEG recordings were preprocessed using the Brain Vision Analyzer (Version 2.1; Brain Products) after file format conversion via BESA Research (Version 7.0). EEG data were preprocessed as described in Helakari et al. (2022). In brief, gradient artifact and ballistocardiographic (BCG) artifact corrections were obtained with the average artifact subtraction method (Allen et al., 1998, 2000). Sleep scoring was performed by two experienced specialists via the standard 10–20 system in 30 s epochs according to AASM guidelines for clinical sleep studies (American Academy of Sleep Medicine, 2017). EEG epochs were scored as awake, N1 (light sleep), N2 (intermediate sleep with sleep spindles and/or K-complexes), N3 (slow wave sleep) or REM (sleep with rapid eye movements), and the final sleep states were obtained by consensus.

The MREG signal was analyzed in two-minute segments, since all subjects had continuous N1 or/and N2 sleep stages for at least this long. For awake data, we chose a two-minute period from the beginning of the scan to ensure high vigilance state. For sleep data, the two-minute period was chosen when at least three quarters of subsequent 30 s epochs were scored as either N1 or N2 stage sleep. If there were many such options, the criteria were: (1) two-minute period from the middle or the end of the stage, to confirm as stable as possible sleep stage, and (2) EEG contained as few artefacts as possible (30 s epoch data with no artefacts were chosen in the preference to the data that included artefacts), thus confirming the sleep stage and minimizing motion artefacts. In the end, we obtained N1 sleep in 13 sleep deprived and 8 non-sleep deprived subjects and N2 sleep in 10 sleep deprived and 4 non-sleep deprived subjects.

### MREG signal spectral entropy analysis

Spectral entropy is an index that has been used in monitoring anesthesia depth (Viertiö-Oja et al., 2004; Vakkuri et al., 2005), sleep (Mahon et al., 2008) and brain activity in epilepsy patients (Bruzzo et al., 2008; Urigüen et al., 2017; Helakari et al., 2019). Previous work has shown that the magnitude of spectral entropy value depends on sleep stage, and type of anesthetic agent. In our prior work (Helakari et al., 2022), we showed that spectral entropy decreased significantly in VLF and respiratory frequency ranges during NREM sleep stages 1–2, indicating that spectral entropy might suffice to separate different brain states.

The spectral entropy method treats the normalized fast Fourier transform (FFT) power spectrum as a probability distribution, which is used to calculate the Shannon entropy (Shannon, 1948). A pure sine wave therefore has a spectral entropy value of 0, and noisy, complex signals have spectral entropy values that approach unity. The spectral entropy value is not only dependent on the mix of frequencies existing in the signal, but also the magnitude of the frequencies in the power spectrum (Anier et al., 2012). If one frequency peak dominates or is relatively stronger compared to others, the spectral entropy value tends to decrease. Some authors suggest that spectral entropy does not describe regularity of the signal so much as its sinusoidality (Jäntti et al., 2004).

We performed the entropy analyses using FSL, AFNI and MATLAB (version R2021b). As in our previous study, we sought to calculate spectral entropy for separate frequency ranges in the MREG signal.

Thus, we filtered the data to full band (0.01–5 Hz), VLF 0.01–0.08 Hz, respiratory (individual, between 0.08 and 0.49 Hz), and cardiac (individual, between 0.51 and 1.56 Hz) frequency ranges using AFNI *3dTProject* command. The observed VLF and respiratory pulsations in the brain tissue are related to T2* BOLD susceptibility changes (Wise et al., 2004; Birn et al., 2006; Kiviniemi et al., 2016) while cardiac pulsations are due to spin phase effects (Von Schulthess and Higgins, 1985) or steady state free precession (SSFP) (Duyn, 1997). The observed changes in CSF are due to T2 inflow or outflow effects (Von Schulthess and Higgins, 1985; Hennig et al., 2021).

Then, we calculated a single spectral entropy value for each two-minute time series (awake, N1 and N2 sleep) of filtered data (FB, VLF, respiratory and cardiac) with MATLAB using the *pentropy*, which produced a spatial map with voxel values ranging between 0 and 1.

### MREG spectral power analysis

Power spectral analysis has been successfully used to separate different physiological phenomena occurring in separate frequency ranges (Duff et al., 2008; Huotari et al., 2019; Raitamaa et al., 2021). We calculated a FFT power density map with the AFNI *3dPeriodogram* for each two-minute (1,200 timepoints) epoch of MREG data. Based on this data length, FFT was conducted with 2048 bins such that 1,024 bins corresponded to the 0–5 Hz frequency range. VLF, respiratory and cardiac FFT ranges were separated from spectral power maps using the individual frequency ranges that were obtained from EtCO_2_ and SpO_2_ signals with *fslroi* in FSL. Summed power over each frequency range was calculated using AFNI *3dTstat*. In the text, we refer to summed power with the term *spectral power*.

### MREG brain tissue type separation

Next, we wanted to investigate whether the spectral entropy and power sum differences corresponded to tissue types of the brain. From spectral entropy and power sum maps, we separated CSF, GM and WM using MNI152 standard bin masks. In addition, we separated 4th ventricle similarly as used in Kananen et al. (2022).

### MREG comparison between sleep deprived and non-sleep deprived subjects

**Figure 1:**
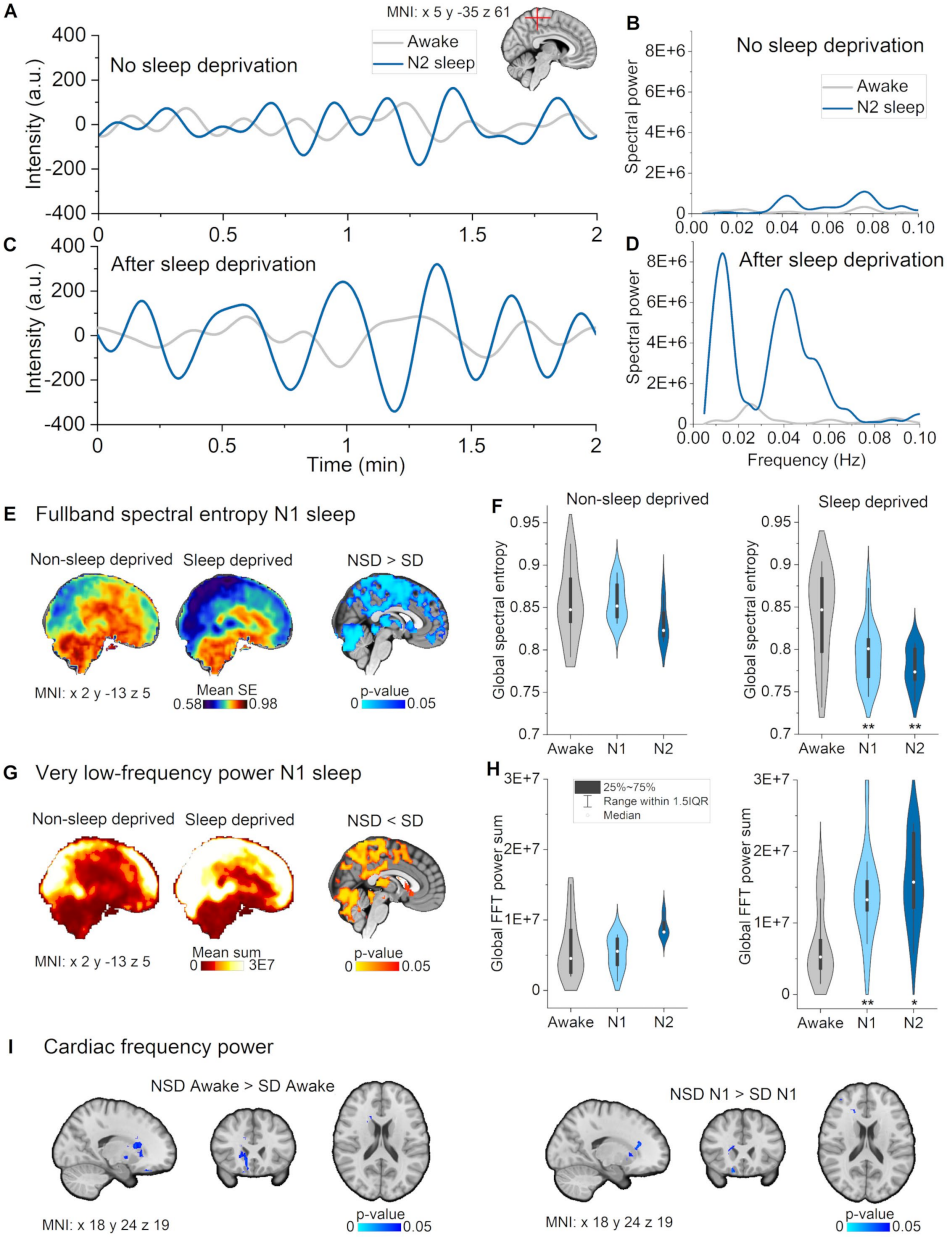
Full band spectral entropy decrease is explicable by increased vasomotor pulsations. **(A)** Vasomotor pulsations during awake and N2 sleep in a representative non-sleep deprived subject. **(B)** Spectral power during awake and N2 sleep in non-sleep deprived subject. **(C)** Vasomotor pulsations during awake and N2 sleep from sleep deprived subject. **(D)** Spectral power during awake and N2 sleep from sleep deprived subject. **(E)** Full band spectral entropy (0.01–5 Hz) mean maps of non-sleep deprived (left) and sleep deprived subjects (right), and respective statistical differences between datasets (*p* < 0.05). **(F)** Global spectral entropy decreased in non-sleep deprived subjects only in N2 sleep, but was lower in sleep deprived subjects in N1 and N2 sleep compared awake. **(G)** Very low-frequency (VLF, 0.01–0.08 Hz) mean spectral power maps of non-sleep deprived (left) and sleep deprived subjects (right), and, respectively, statistically significant differences between datasets (*p* < 0.05). **(H)** Global FFT power in VLF increased in non-sleep deprived subjects only in N2 sleep, but in sleep deprived subjects in N1 and N2 sleep as compared to awake. **(I)** Spectral power in cardiac frequency was significantly decreased in sleep deprived subjects in the awake and N1 data recordings in nucleus caudatus and fronto-basal cortex. **(B,D)** Paired sample *t*-test within the dataset: awake versus N1 and N2: *p < 0.05, **p < 0.01. Subjects: Non-sleep deprived subjects while awake (*n* = 10), N1 (*n* = 8) and N2 (*n* = 4), sleep deprived subjects awake (*n* = 13), N1 (*n* = 12) and N2 (*n* = 10). SD, sleep deprived; NSD, non-sleep deprived.

Even a single night of sleep deprivation increases beta amyloid burden (Kang et al., 2009; Shokri-Kojori et al., 2018). Furthermore, EEG delta power increases after sleep deprivation in comparison to regular sleep (Hubbard et al., 2020). Therefore, we investigated whether the MREG results of sleep deprived subjects (1) were different from non-sleep deprived subjects (2). We made the comparison between awake data of datasets 1 and 2 (12 versus 8 subjects) for negative control to identify possible subject-related changes that had no bearing on sleep deprivation, and similarly for N1 sleep (12 versus 8 subjects) in all frequency ranges of spectral entropy and power sum. The N2 comparison was unreliable due to inadequate sample size (10 versus 4 subjects); see Figures 1F,H for mean global values in N2 sleep.

### Statistical analysis

We used the two-sample paired *t*-test model with threshold-free cluster enhancement and family-wise error (false positive) correction in FSL *randomize*, with the default 4,096 permutations for paired analysis. We compared voxel-wise spectral entropy and power maps (awake versus N1 sleep, awake versus N2 sleep), using the data of same subjects in each comparison (21 awake versus 21 N1, 14 awake versus 14 N2). For sleep deprived versus non-sleep deprived subject groups, we used the two-sample unpaired *t*-test model with 5,000 permutations using FSL randomize for multiple comparison correction. Differences between single values in CSF, GM, WM and 4th ventricle were calculated by using a two-sample paired *t*-test model in Origin (version 2020b). Physiological EtCO_2_ and SpO_2_ statistical analyses were performed similarly (Rajna et al., 2019, 2021).

## Results

### EEG sleep stages

Two experienced clinical neurophysiologists performed consensus sleep scoring according to 10–20 system in 30 s epochs, following AASM guidelines for clinical sleep studies (American Academy of Sleep Medicine, 2017). We obtained 10–20 min of sleep data from each subject, from which most of the data were scored as N1 or N2 stage sleep, with one subject experiencing two 30 s epochs of N3 stage sleep (Table 1). We chose to use two-minute data series for the analysis, since all subjects had at least two minutes of continuous N1 or/and N2 sleep stage. Awake eyes open scans consisted 99,5–100% of wakefulness and sleep scans 37,7–53% of N1 sleep and 24,6–37,2% of N2 sleep.

**Table 1:** Amount of sleep (% of epochs calculated from total amount of epochs) for data set 1, sleep deprived subjects and data set 2, non-sleep deprived subjects.

	Data set 1, sleep deprived subjects				
	W	N1	N2	N3	Artifacts
Awake eyes open, *n* = 11	99,5	0,5	-	-	-
Awake eyes closed, *n* = 11	66,4	29,1	4,5	-	-
Sleep scan 1, *n* = 12	16,8	43,7	35,7	0,4	4,0
Sleep scan 2, *n* = 11	24,1	37,7	37,2	-	1,0
	Data set 2, non-sleep deprived subjects				
Awake eyes open, *n* = 10	100,0	-	-	-	-
Sleep scan, *n* = 10	17,0	53,0	24,6	1,0	4,0

Data set 1 consisted of one 10-min awake eyes open and one 5-min eyes closed scans and two 10-min sleep scans. Data set 2 consisted of one 10-min eyes open awake scan and one 10-min sleep scan. W = awake, N1 = NREM stage 1, N2 = NREM stage 2, N3 = NREM stage 3, Artifacts (scoring not possible due to EEG artifacts).

### Physiological EtCO_2_ and SpO_2_ signals were used to determine respiratory and cardiac frequency ranges

Individual minimum, maximum, and peaking values for respiration and heart rates were evaluated from power spectra of EtCO_2_ or respiratory belt and SpO_2_ signals. We chose respiratory and cardiac minimum and maximum values such, that the power spectra met an adequate noise level, and to exclude harmonics or heterodynes in the frequency range. Average ± standard deviation (SD) of the results in the two groups are presented in Table 2. Subsequently, we used individual frequency ranges for further analysis. We chose the VLF range with a lower cut-off at 0.01–0.08 Hz such that it did no overlap with the respiratory frequency range. Data of sleep deprived and non-sleep deprived subjects are presented for the sake of comparison (no statistically significant differences between datasets (1) and (2), 12 versus 10, Table 2).

**Table 2:** Minimum, maximum, and highest peaking values of physiological EtCO_2_ or respiratory belt and SpO_2_ spectra during awake and sleep scans.

	EtCO_2_ or resp. belt (Hz)			SpO_2_ (Hz)		
	Min	Peak	Max	Min	Peak	Max
	(1) Sleep deprived subjects					
Awake scan *n* = 12	0.15 ± 0.06	0.27 ± 0.05	0.40 ± 0.06	0.87 ± 0.16	1.03 ± 0.18	1.23 ± 0.23
Sleep scan 1 *n* = 12	0.14 ± 0.04	0.25 ± 0.04	0.35 ± 0.04	0.77 ± 0.12*	0.94 ± 0.17*	1.16 ± 0.22
Sleep scan 2 *n* = 11	0.14 ± 0.06	0.24 ± 0.06	0.35 ± 0.08	0.79 ± 0.12	0.97 ± 0.17	1.19 ± 0.22
	Respiratory 0.08–0.49 Hz and cardiac range 0.51–1.56 Hz.					
	(2) Non-sleep deprived subjects					
Awake scan *n* = 10	0.16 ± 0.06	0.27 ± 0.06	0.38 ± 0.07	0.82 ± 0.13	1.00 ± 0.11	1.20 ± 0.15
Sleep scan *n* = 10	0.17 ± 0.06	0.29 ± 0.05	0.36 ± 0.06	0.83 ± 0.06	1.00 ± 0.11	1.07 ± 0.37
	Respiratory 0.08–0.47 Hz and cardiac range 0.66–1.47 Hz.					

There were no statistically significant differences between sleep deprived and non-sleep deprived subjects. Statistical differences between Awake and Sleep scans, average ± standard deviation, Awake compared to Sleep scans: *p* < 0.05*.

### Sleep deprivation was confirmed using the smart ring data

In this study we compared two data sets from which the first one (data set 1) consisted of sleep deprived subjects. We used the smart ring to confirm the sleep deprivation of the subjects. For a detailed description, please see Helakari et al. (2022). Supplementary Table 2 consists of individual duration of deprivation and amount of sleep. Duration of sleep deprivation (Average ± STD over subjects) was 24.13 ± 1.42 h, and amount of sleep before Awake scan session 7.68 ± 1.36 h (normal night sleep) and amount of sleep before Sleep scan session 11.27 ± 21.02 min (sleep deprivation night). This data indicates that before Awake scan, subjects had sufficient amount of sleep, and before Sleep scan they clearly stayed awake most of the night.

### Sleep deprivation decreased full band spectral entropy and increased the power of vasomotor pulsation

We first asked whether the three physiological pulsations were affected by sleep deprivation. We calculated spectral entropy to study the stability of the pulsations, and quantified spectral power to obtain the strength of the pulsations for awake, N1, and N2 data for both participant groups. For statistical voxel-wise analysis, we used the N1 sleep epochs, which were sufficiently abundant to serve for this comparison (8 non-sleep-deprived versus 12 sleep-deprived subjects).

**Figure 2:**
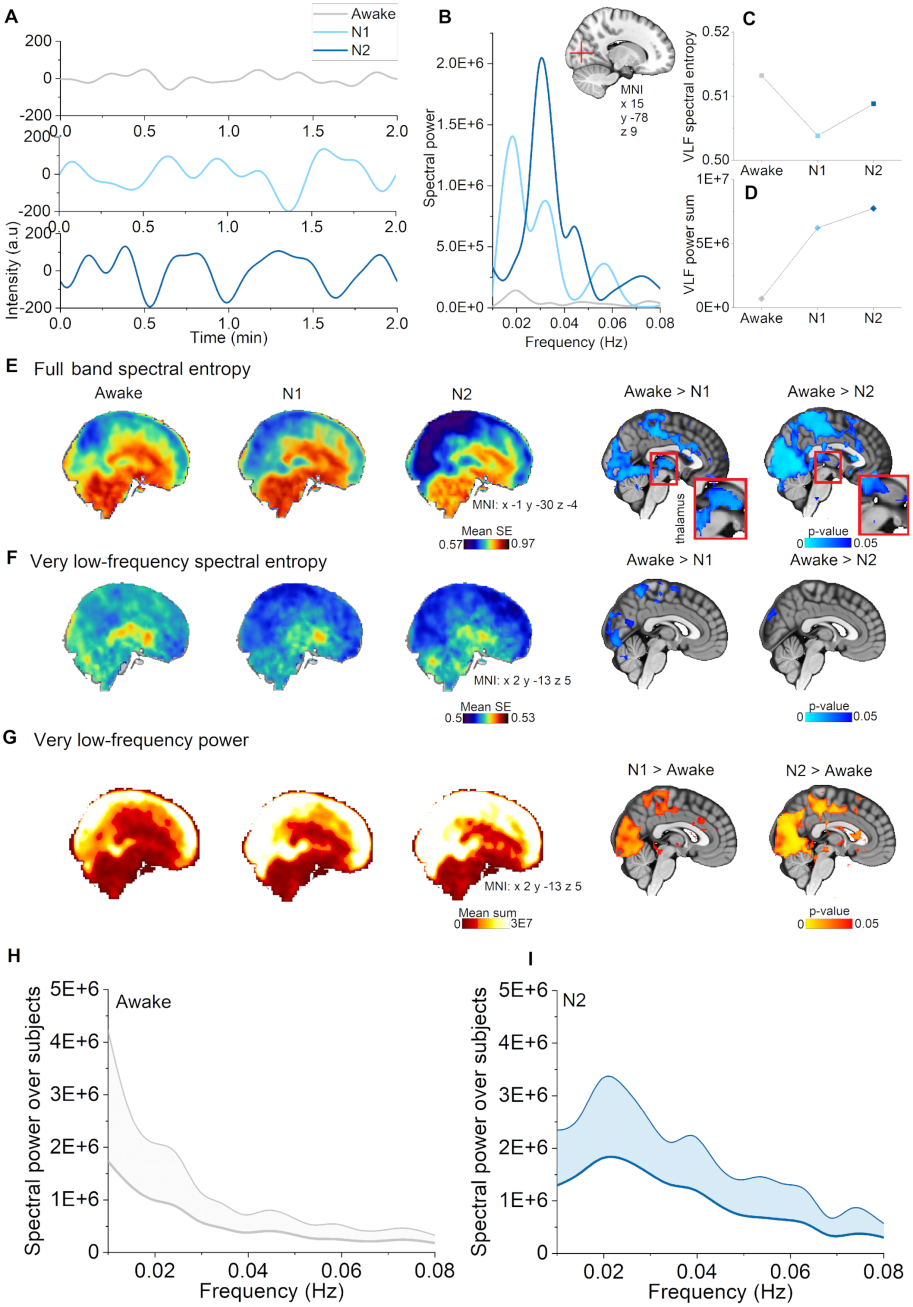
Vasomotor pulsations intensified during N1 and N2 sleep stages compared with awake. **(A)** Very low-frequency (VLF) pulsations (0.01–0.08 Hz) increased and harmonized especially during N2 sleep (representative signals from visual cortex, non-sleep deprived subject). **(B)** VLF power spectra during awake, N1, and N2 sleep from the pulsations in A. **(C)** VLF spectral entropy in a voxel within visual cortex decreased during N1 and N2 sleep stages compared to wakefulness. **(D)** VLF spectral power in a voxel within visual cortex increased during N1 and N2 sleep. **(E)** Mean spectral entropy maps of full band (0.1–5 Hz) and in the right column the corresponding, statistically significant differences between awake and sleep stages (*p* < 0.05). **(F)** Mean spectral entropy maps of VLF (0.01–0.08 Hz) and in the right column the corresponding, statistically significant differences (*p* < 0.05). **(G)** Mean spectral power maps of VLF and in the right column, the corresponding statistically significant differences. Subjects: awake (*n* = 23), N1 (*n* = 21), and N2 (*n* = 14) sleep. **(H)** Awake whole brain VLF power spectrum (mean ± STD) across subjects [*n* = 14, same subjects as in **(I)**], and **(I)** N2 sleep whole brain VLF power spectrum (mean ± STD) across subjects (*n* = 14).

We detected statistically significant group differences in N1 sleep in full band spectral entropy (non-sleep deprived > sleep deprived, *p* < 0.05, *n* = 8 versus 12) and VLF spectral power (non-sleep -deprived < sleep deprived, *p* < 0.05, *n* = 8 versus 12, Figure 2). VLF results had a wide spatial overlap with full band results, indicating that increased VLF power could largely explain the spectral entropy results. More specifically, spectral entropy changes were more prominent across broad brain regions, extending from posterior to anterior cortex, and including superior cerebellum, the lateral ventricles and the thalamus (Figures 2E,F). Significant VLF differences were found in bilateral posterior brain regions, including visual-, precuneus- and somatosensory cortex, posterior cingulate gyrus, cerebellum, the lateral ventricles, and thalamus (Figures 2G,H).

Additionally, we observed higher cardiac power during awake (normal eyes open rest) and in N1 sleep in the non-sleep deprived versus the sleep deprived group in nucleus caudatus and fronto-basal cortex (*n* = 8 versus 12, Figure 2I). Since these changes occurred in the awake comparison, those could not be attributed to sleep deprivation *per se*, but rather reflected differences between the two subject groups. Overall, these cross-sectional results indicate that sleep deprivation selectively promoted vasomotor pulsations.

### Full band spectral entropy decreases in specific regions depending on the sleep stage

Classical fMRI studies have tended to analyze the full extent of the available BOLD signal by using methods such as amplitude of low frequency fluctuations (ALFF) (Gao et al., 2015). To investigate whether the overall pulsatility of the brain becomes more stable during sleep versus awake states, we calculated full band (0.01–5 Hz) spectral entropy of MREG. Here, we found that full band spectral entropy decreased as a function of sleep depth across N1 and N2 stages in the posterior sensory brain regions including visual, auditory, somatosensory cortices, cingulate gyrus, orbitofrontal and ventromedial prefrontal cortex (Figure 1E). Interestingly, we saw decreased spectral entropy in thalamus and upper brainstem in N1, but not in N2 sleep. In general, entropy changes encompassed larger regions occurring in N2 than in N1, with the difference that in N2 sleep, the changes extended to include the sensory association area, motor cortex, basal forebrain, lower brainstem, and inferior cerebellum. Pulsations assumed a more regular pattern in both N1 and N2 sleep stages.

### Sleep stage analysis revealed that vasomotor pulsations intensified in N1 and N2 sleep

Next, we studied the VLF (0.01–0.08) Hz band to investigate whether changes in full band spectral entropy were explicable by changes in that particular frequency range. We found that spectral entropy of VLF decreased in visual, and somatosensory cortex during N1 sleep, but only in visual cortex during N2 sleep, as compared to awake (Figure 1F). We identified a power increase in larger regions encompassing posterior parts of the brain, especially covering visual and somatosensory cortex, anterior cingulate gyrus and upper cerebellum (Figure 1G), namely in the same regions that displayed a decrease in full band spectral entropy. In addition to broader regional changes, we found increases in the sensory association area, motor cortex, auditory cortex, upper brainstem, and the lateral ventricles during N2 sleep. We present representative examples of pulsations, power spectra, and spectral entropy in Figures 1A–D. Global VLF power spectrum over subjects in N2 sleep demonstrates peak in 0.02 Hz (Figure 1I). Presentation of the full band results together with VLF results (Figure 1) make the comparison easier, suggesting that VLF was the main source of full band spectral entropy changes. In our previous study, we similarly showed that full band spectral entropy could separate sleep from wakefulness states (Helakari et al., 2022). In conclusion, VLF pulsations intensified in N1 sleep compared with awake, and encompassed broader brain areas in N2 sleep.

### Respiratory pulsations intensified and harmonized selectively only in N2 sleep

**Figure 3:**
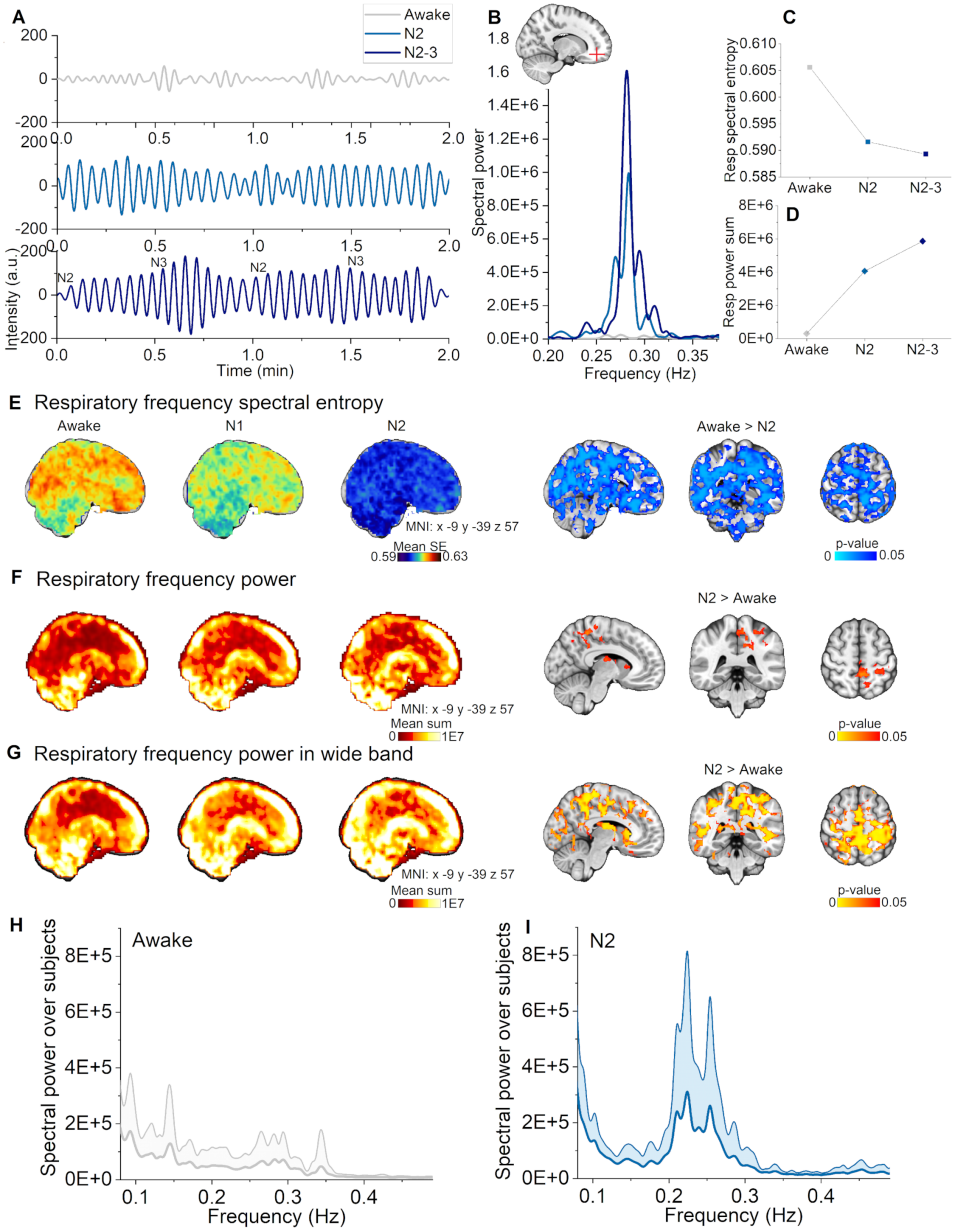
Respiratory pulsations increased and harmonized during N2, but not in N1 sleep. **(A)** Representative example of respiratory pulsations during wakefulness, N2, and N2-3 (half of the signal scored as N3) sleep from the frontal cortex (non-sleep deprived subject). **(B)** Example spectra in the respiratory frequency band from the pulsations in **(A)**. **(C)** Spectral entropy of respiratory pulsations decrease during N2 and N2-3 sleep calculated from the pulsations in **(A)**. **(D)** Spectral power increased during N2 and N2-3 sleep calculated from the pulsations in **(A)**. **(E)** Respiratory pulsation spectral entropy mean map across subjects [left column, awake (*n* = 23), N1 (*n* = 21) and N2 (*n* = 14)]. Statistically significant differences in spectral entropy between awake and N2 sleep were found for whole brain (right column). **(F)** Primary respiratory power mean map across subjects [left column, awake (*n* = 23), N1 (*n* = 21) and N2 (*n* = 14)]. Statistically significant differences of spectral power between awake and N2 sleep (right column). **(G)** Wide band respiratory power mean map over subjects in the wide frequency band [0.08–0.49 Hz; left column, awake (*n* = 23), N1 (*n* = 21) and N2 (*n* = 14)]. Statistically significant differences in spectral power between awake and N2 sleep in the wide frequency band (right column). **(H)** Awake whole brain respiratory power spectrum (mean ± STD) across subjects [*n* = 14, same subjects as in **(I)**], and **(I)** N2 sleep whole brain respiratory power spectrum (mean ± STD) across subjects (*n* = 14).

Next, we tested the proposition that spectral entropy in the respiratory band (individual range 0.08–0.49 Hz) would decrease and power increase in N1 and N2 sleep stages versus awake. Indeed, respiratory spectral entropy decreased over broad brain regions in N2 sleep (Awake > Sleep, *p* < 0.05, Figure 3E), but not during N1 sleep. The highest statistical differences were found in the ventromedial prefrontal cortex and in visual cortex. Spectral power increased significantly in somatosensory cortex and lateral ventricles only in N2 sleep (Awake < N2, *p* < 0.05, Figure 3F). As in our previous study (Helakari et al., 2022), we found larger changes in the whole respiratory frequency range, when comparing awake data to N2 sleep across a wide frequency range (0.08–0.49 Hz). The awake versus N2 contrast showed differences in larger brain regions than when using an individual frequency range, encompassing visual, somatosensory and auditory cortices, part of the cingulate gyrus, the lateral ventricles, basal forebrain, thalamus, cerebellum and midbrain (Figure 3G). It seems that using individual frequency range rather than wide range over group, spectral entropy results tend to be emphasized while power results diminish. We note further considerations about the differences in the discussion section. Representative examples of pulsations, spectra, and spectral entropy, and power values can be seen in Figures 3A–D. Global power spectra over subjects during awake and N2 sleep are presented in Figures 3H,I. It can be noticed, that power spectra shape tends to center in narrower band during N2 sleep when compared awake, which we suggest to reflect more regular pattern in the pulsation as seen in Figure 3A. Respiratory pulsation changes were selective for N2 sleep, perhaps indicating physiological functions that are operational in intermediate sleep, but not yet evident in the early sleep transition.

### Cardiac pulsations are intensified in N1 and N2 sleep

**Figure 4:**
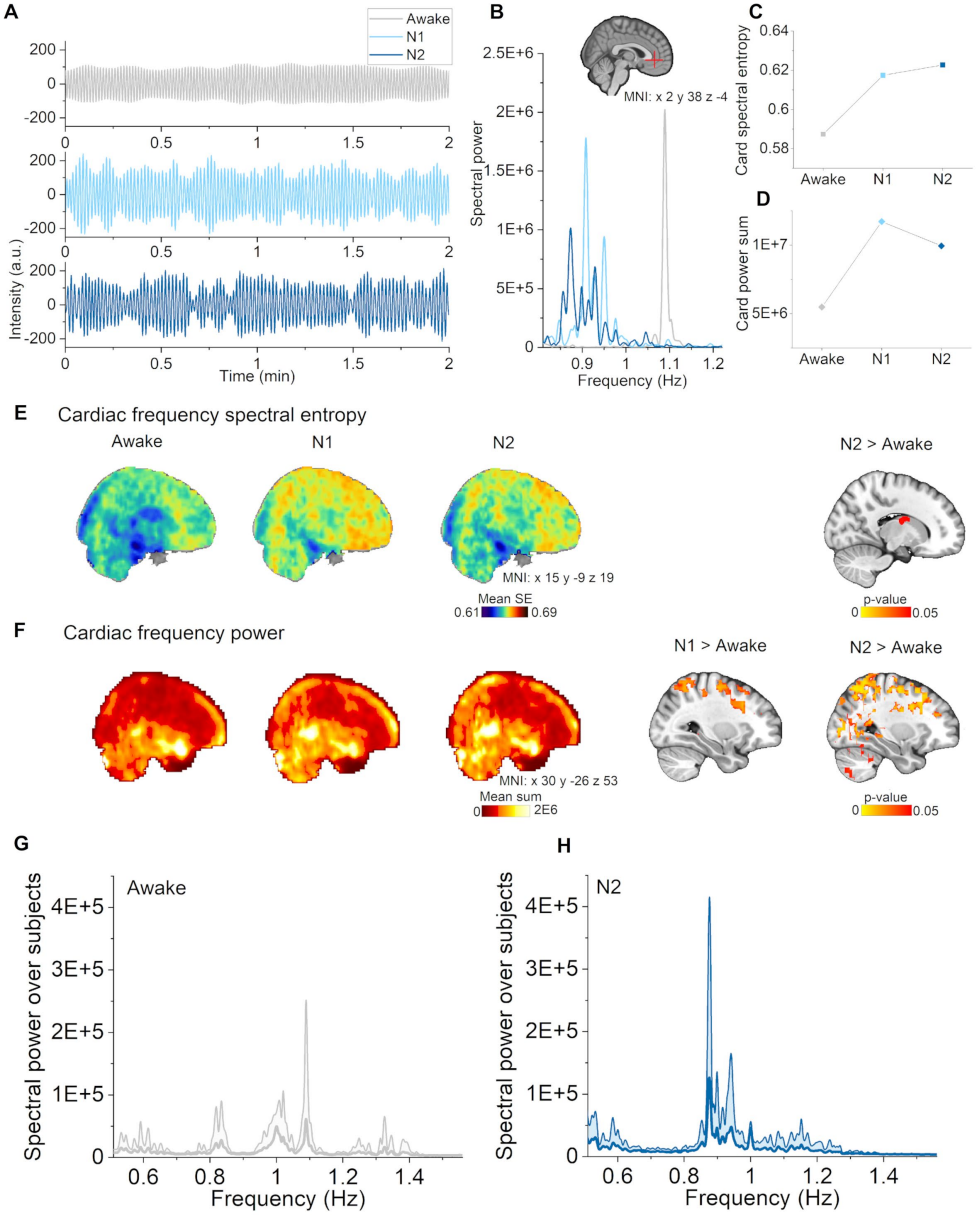
Cardiac pulsations increased in N1 and N2 sleep. **(A)** Cardiac pulsations (individual range 0.82–1.22 Hz) during waking, and in N1 and N2 sleep located in the anterior cerebral artery (non-sleep deprived subject). **(B)** Cardiac power spectra during awake, N1 and N2 sleep in the anterior cerebral artery. **(C)** Cardiac spectral entropy increased in N1 and N2 sleep in the anterior cerebral artery. **(D)** Cardiac spectral power increased in N1 and N2 sleep in the anterior cerebral artery. **(E)** Mean spectral entropy maps of cardiac pulsations (individual between 0.51–1.56 Hz, left) and statistical differences between awake and sleep stages (right). **(F)** Mean spectral power maps of cardiac pulsations and (right column) differences between awake and sleep stages. Subjects: awake (*n* = 23), N1 (*n* = 21) and N2 (*n* = 14) sleep. **(G)** Awake whole brain cardiac power spectrum (mean ± STD) across subjects [*n* = 14, same subjects as in **(H)**], and **(H)** N2 sleep whole brain cardiac power spectrum (mean ± STD) across subjects (*n* = 14).

Next, we asked whether cardiac pulsation changes also occur across different sleep stages. In spectral power analysis, cardiac pulsations behaved similarly as vasomotor pulsations, i.e., that there was an increase in cardiac power in N1, with more widespread increases in N2 (Figure 4F). The N1 differences mainly occurred in somatosensory cortex and the sensory association area, where there were additional differences during N2 in cingulate gyrus, motor and visual cortices, and in cerebellum (Figure 4F, right column).

Instead of decreasing, spectral entropy of cardiac pulsations was higher in the lateral ventricles (Figure 4E, right column) with increasing sleep stage. In general, the mean maps showed a trend towards increasing spectral entropy also in wider brain regions (Figure 4E, left column). Example of cardiovascular pulsations in the anterior cerebral artery along with spectra, spectral entropy and power values are presented in Figures 4A–D. These findings indicate that cardiac pulsations became stronger but more variable as the subjects transitioned from wake to N1 and finally to N2 sleep.

### Pulsation changes are dependent on the brain tissue

**Figure 5:**
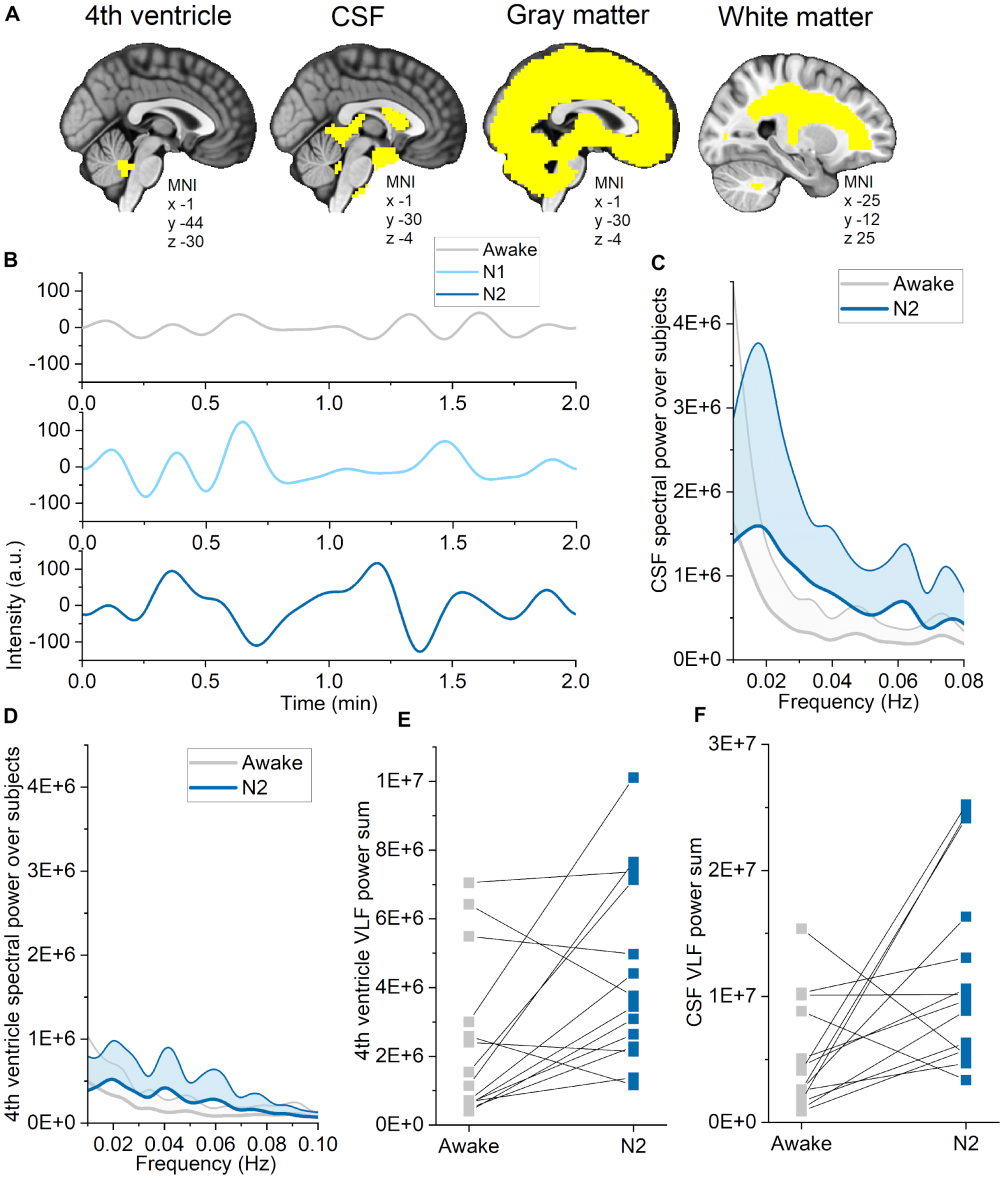
Pulsations in CSF and the 4th ventricle intensified in N2 sleep. **(A)** Standard MNI152 masks for cerebrospinal fluid (CSF), gray matter and white matter were used to calculate single spectral entropy and power values for each tissue types during wakefulness, and in N1 and N2 sleep. The 4th ventricle region of interest was identified based on Kananen et al. (2022). **(B)** Example of very low-frequency (VLF) CSF signal during wakefulness, and in N1 and N2 sleep from one subject (sleep deprived). **(C)** VLF CSF spectral power across subjects (average ± STD) during wakefulness (gray) and N2 sleep (blue). **(D)** VLF 4th ventricle spectral power across subjects (average ± STD) during wakefulness (gray) and N2 sleep (blue). **(E)** Sum of VLF spectral power from the 4th ventricle during wakefulness and N2 sleep from representative individuals (*n* = 14). **(F)** Sum of VLF spectral power from CSF during wakefulness and N2 sleep from representative individuals (*n* = 14).

Next, we investigated whether pulsation changes are dependent on the brain tissue type or region. We segmented CSF, WM, and GM using standard MNI152 masks, and the 4th ventricle as described elsewhere (Kananen et al., 2022) as regions of interest (Figure 5). Spectral power was already increased in N1 sleep, and spatially broader in N2 sleep, including all three pulsations and more tissue types (Table 3). Spectral entropy of VLF (N1) and respiratory pulsations (N2) were lower in most tissue types, while cardiac spectral entropy increased only in the CSF compartment. These indices were affected in the 4th ventricle and CSF only in N2, but not in N1 sleep. During N2 sleep, 4th ventricle CSF pulsation peaks across all subjects were observed at 0.02, 0.04, and 0.06 Hz. While using a CSF pulsation mask covering main regions of the CSF, the peak was clearly located at 0.02 Hz and to lesser extent at 0.06 Hz. These findings support the previous literature that CSF flow increases during N2 sleep (Fultz et al., 2019), with the difference that their peaking value in CSF was presented in 0.05 Hz.

**Table 3:** Spectral entropy and power differences in N1 and N2 sleep stages compared to waking.

	Spectral entropy		Spectral power	
	N1	N2	N1	N2
VLF	GM, WM ↓	-	GM ↑	CSF, 4th, GM, WM ↑
RESP	-	4th, GM, WM ↓	-	GM, WM ↑
CARD	-	CSF↑	GM, WM ↑	4th, GM, WM ↑

Only statistically significant areas are reported (*p* < 0.05, paired *t*-test). Spectral entropy was mainly lower, and power higher during N1 and N2 sleep as compared to waking. ↓ corresponds to decreased value in N1 or N2 sleep versus awake and ↑ corresponds to increased value.

## Discussion

In this study, we investigated the effect of acute sleep deprivation and electrophysiologically different sleep stages on physiological brain pulsations detected by fast fMRI in young healthy subjects. Our major observation was that sleep deprivation selectively increased the amplitude and incidence of vasomotor pulsations during N1 sleep, without any effect on cardiorespiratory pulsations. This observation is in line with a previously reported association between sleep pressure and slow delta wave activity. The stability of the full band and respiratory pulsations, and the power of all pulsations, increased as a function of sleep depth, i.e., sleep stage, indicating that sleep staging not only impacts neural activity (EEG, AASM criteria), but also effects physiological pulsations. CSF and 4th ventricle pulsations intensified only in N2 sleep, indicating enhanced fluid clearance during intermediate sleep. These cross-sectional observations show that physiological pulsations are dependent on vigilance level and sleep stage.

### Sleep after sleep deprivation intensified vasomotor pulsations

Our analysis showed that vasomotor, but not cardiorespiratory pulsations intensified during N1 stage after sleep deprivation compared with subjects who had not been exposed to sleep deprivation. To our knowledge, this is the first evidence that increased sleep pressure as associated with increases in homeostatic physiological process beyond the well-known effects on the slow delta wave power to EEG. These observations suggest that sleep pressure can potentiate a house-keeping function of sleep with respect to solute clearance.

A dominant driver for sleep-related VLF brain activity might arise in the locus coeruleus, where 0.03 Hz fluctuations in the firing rate of norepinephrine neurons control the microarchitecture of NREM sleep in mice (Kjaerby et al., 2022). In addition, monkey and human research indicate that the basal nucleus may be also be involved in controlling widespread vasomotor waves during sleep (Chang et al., 2016; Liu et al., 2018). Other studies have shown that sleep deprivation, reduced vigilance level, and early sleep increase VLF fluctuations in the brain, most prominently in the primary visual cortex (Fukunaga et al., 2006; Horovitz et al., 2008; Gao et al., 2015; Chang et al., 2016; Liu et al., 2018). Thus, vasomotor pulsations may be influenced by sleep pressure and homeostasis as presented in the original Borbely two-process model (Borbély, 1982; Borbély et al., 2016). Present results indicate that changes in vasomotor pulsations are dependent on sleep history, vigilance level, and sleep stage.

Our findings showed that, average spectral entropy and power changes clearly occurred in N1 and N2 stage versus awake state in sleep deprived subjects, whereas only circumscribed changes occurred in non-sleep deprived subjects, and then only in the intermediate N2 stage (Figures 2F,H). These results indicate that, although the sleep epoch was scored electrophysiologically as stage N1, the physiological pulsations varied within the same stage depending on the vigilance state. Because the sleep stages were scored in 30 s epochs according to AASM rules, sleep characteristics may have fluctuated during that time, potentially causing some of observed differences. However, it might also be, that sleep pressure is driven in part by metabolic waste accumulation, such that sleep deprivation evokes a compensatory increase in solute transport. The causal mechanisms underlying such a conjectural control system remain to be established.

The above speculations aside, delta power clearly increases upon sleep deprivation (Marzano et al., 2010; Hubbard et al., 2020) with spatial overlap with electrophysiological changes reported in our previous study (Helakari et al., 2022). How sleep pressure is linked to vasomotor frequency is presently unknown. The observed changes after sleep deprivation were prominent in thalamus, cerebellum, and broad regions of cerebral cortex. We suggest present results highlight the need to consider that remarkable physiological changes in pulsatility arise indeed within N1 sleep even in healthy subjects, and to an extent depending on the vigilance state. Vasomotion seems be the first one to increase the sleep related clearance in healthy subjects, but it’s significance to the tracer clearance should be further studied either in human or animal experiments. Previous studies using subarachnoid MR contrast convection over hours/days show that sleep deprivation impairs the tracer clearance and even subsequent sleep does not correct situation on NPH patients (Eide et al., 2021,^2022^). It might be, that achieving the full level of metabolic solute transport during sleep, longer sleep cycles and even many well slept nights are needed.

In sleep stage analysis, we showed that vasomotor frequency pulsations had already increased in the N1 stage of sleep transition, which corresponds to light sleep or drowsiness. The main changes were found in visual cortex and somatosensory cortex. One striking observation is that, as the subjects transitioned from lighter to intermediate N2 stage sleep, the regions with vasomotor pulsations expanded to include broader brain regions. Recent article by Song et al. (2022) supports our finding that VLF pulsations (<0.1 Hz) occur already in the light sleep. Interestingly, they found that higher frequencies (0.1–0.2 Hz) are prevalent in N3 sleep, but due to the lack of N3 sleep in our data, we are unable to confirm this finding at this point. Whereas in N1 sleep, the vasomotor changes were mainly confined to GM and WM, in N2 sleep the changes across nearly the whole brain and in the CSF. While the changes in 4th ventricle and extra-axial CSF were first evident in N2 sleep, the results support the idea that actual sleep instead of pure drowsiness is a necessary requirement for elevated CSF flow and solute transport (Hablitz and Nedergaard, 2021). This may indicate that while changes in VLF arise in part from neuronal activity changes (e.g., in relation to delta waves), in the transition to deeper sleep stages, fluid and tissue pulsatility may have a higher vascular dependence. Supporting our results, also sympathetic nervous system contribution, more specifically K-complexes or vascular responses driven by arousals during N2 sleep, have been connected to large-amplitude fMRI fluctuations (Özbay et al., 2019; Picchioni et al., 2022). It seems plausible, that these effect may cause vasomotion and further contribute ton CSF flow in the perivascular spaces (Bojarskaite et al., 2023; Holstein-Rønsbo et al., 2023). As CSF flow rate correlates with intracerebral blood volume changes (Fultz et al., 2019), it might be supposed that blood volume could directly affect CSF pulsations.

### Respiratory pulsations changes are specific to N2 sleep

The present findings that respiratory pulsations harmonized across the whole brain during N2 sleep could well have a physiological basis. Previous work shows that, as sleep stabilizes after its onset, heart rate declines and respiratory variability assumes a more regular pattern (Malik et al., 2012). We found only two epochs of N3 sleep in the whole dataset, but this limited data suggested that respiratory pulsations became even more balanced and more powerful in the transition from N2 to N3 sleep (Figures 3A–D). Further studies employing the fast fMRI methods might establish better the pulsation changes during N3 sleep and extending REM sleep.

When brain-wide spectral entropy decreased during sleep, and the power of the pulsations increased across somatosensory and visual cortices. These observations are consistent with our previous findings, albeit confined to smaller regions than in the earlier work (Helakari et al., 2022). We herein selected an individual frequency range to focus specifically on the phenomenon studied (i.e., respiration), which may in some cases have limited the frequency to an excessively narrow band, given that the appropriate range may vary between different voxels. To make a clearer comparison to our previous study, we also undertook the calculations with a wide frequency range, whereupon the sleep stage dependent changes occurred in broader brain regions, as expected. In conclusion, both approaches to setting the frequency bandwidth were successful, but with differing limitations: a wide spectral range may include variability due to noise, respiratory harmonics, and modulatory heterodyne peaks (Raitamaa et al., 2021), whereas a narrow or tailored frequency window will tend to result in lesser signal.

During sleep, respiration is fully under control of the brainstem and complex mechanisms regulated by mechanical (e.g., stretch receptors) and chemical (e.g., chemoreceptors) information (Sowho et al., 2014). A recent study indicated that voluntary yogic deep abdominal breathing (pranayama) in conjunction with associated cardiac changes accelerated CSF flow when compared to spontaneous breathing (Yildiz et al., 2022). In addition, muscle activity in the diaphragm during sleep, often designated abdominal breathing, increases the load on parasternal intercostal muscles (Yokoba et al., 2016).

Conceivably, breathing during sleep and pranayama present similar physiological approaches to increase CSF flow. In line with this conjecture, Picchioni et al. (2022) have recently shown that N1-2 sleep has similar effects on CSF flow as did self-controlled deep breathing. Our present findings suggest that respiratory pulsation increases in the brain during N2 sleep are a driver for increased CSF flow.

### Cardiac pulsations became stronger and more variable in N1 and N2 sleep

In this study we showed that spectral entropy of cardiac pulsations tended to increase in the anterior parts of the brain in N1 and N2 sleep as compared with waking (Figure 4E). Statistically significant differences were found only in the lateral ventricles during N2 sleep. However, we note that beat-to-beat heart rate variability (HRV) increases during NREM sleep (Sattin et al., 2021). As we know that higher spectral entropy corresponds to a higher number of frequencies in the spectrum, the cardiac entropy changes may well reflect HRV changes as seen in the anterior cerebral artery (Figures 4A,B). In future research, one should investigate cardiac brain pulsations during sleep using the conventional HRV parameters.

In addition to increased spectral entropy, cardiac pulsation power increased in cerebral cortex in N1 and N2 sleep. In our previous report, the corresponding changes occurred in smaller region (Helakari et al., 2022), suggesting that cardiac changes may be more sensitively discovered when applying a more limited frequency range. As our findings for respiratory and cardiac pulsations were in overlapping brain regions, there may be cardiorespiratory modulation of these frequencies (Raitamaa et al., 2021).

Interestingly, the brain regions showing increased cardiac power during sleep were in the posterior regions, while spectral entropy tended to increase in the anterior brain regions. As yet we have no explanation for this compelling result. Although power increase often parallels with decreased spectral entropy as in vasomotor and respiratory ranges, in cardiac frequency we saw increase in both values. Spectral shape corresponding to increase in entropy during N1 and N2 sleep is represented in Figure 4B, supporting the increase in HRV. In general, our finding of increased cardiac pulsations during sleep is consistent with glymphatic studies showing that cardiovascular pulsations drive brain fluid clearance (Iliff et al., 2013; Mestre et al., 2018) Sleep spindles have onset in N2 sleep, and may be coincident with low frequency heart rate oscillations in somatosensory cortex (Lecci et al., 2017).

## Limitations

There are several limitations in this study. Sleep measurements in the MRI scanner is unfortunately challenging due to movement restrictions, scanner noise and discomfort imposed by the measuring devices, e.g., EEG cap. Thus, falling asleep and staying asleep may be difficult. Because of this the sleep quality may not be as good as at home. Our previous article (Helakari et al., 2022) and also other recent study (Fultz et al., 2019) presented only N2 sleep during EEG-fMRI, and even those who have managed to scan N3 or REM sleep still reported low sleep efficiency (Moehlman et al., 2019; Özbay et al., 2019; Picchioni et al., 2022).

Our study group only included young, healthy subjects and cannot be generalized to a common population. In this study, we did not screen the sleeping patterns nor sleep history of the subjects. Also, our subjects were healthy based on interview, but we did not screen them for sleep disorders or sleep apnea.

After EEG based sleep stage scoring, our sample size in sleep deprivation versus non-sleep deprivation comparison was relatively small. For comparing N1 and N2 sleep stages to wakefulness, we pooled sleep deprived and non-sleep deprived subjects to reach sufficient sample size. Sleep scans for sleep deprivation and non-sleep deprivation groups were done in different time of day, leading to the possibility of confounding circadian effect. However, separate group analysis revealed no changes in cardiorespiratory frequencies between these groups, which supports the idea that sleep-stage results are rather sleep-based than circadian-based.

The sleep stages were fragmented devoid of continuous epochs of sleep stages (awake, N1, or N2) over several minutes. Thus, we were only able to use the best available two-minute length data for the comparison. Epochs with uninterpretable EEG data quality, consisted of artifacts due to motion, insufficient contact of the electrode or sweating, were also removed from the study. Therefore, we were not able to take account the sleep onset or compare the same location in the signal for the sleep. However, from each subjects, we took N1 and N2 data that were in most stabile phase of the stage for strict separation between sleep stages. Also, when considering the length of the data, cutting and combining data from several locations from the signal would not have been ideal way to analyze the data; using continuous signal, although shorter, is more appropriate, and still brings statistically relevant information from the fast sampled fMRI with 1,200 brain volumes per scan. For sleep score information, we used 30 s epochs and therefore, some N1 data may have included more wakefulness than others (when minimum 16 s is N1 sleep, the data is scored N1). In this study, as persued to scan pure resting-state, we did not use any additional tasks such as cued button press response (Fultz et al., 2019; Setzer et al., 2022). We determined the frequency range from whole 10-min data instead of two-minutes. However, as we used individual frequency range, we suggest that carefully limited frequency ranges are sufficient for this analysis.

## Conclusion

The purpose of this study was to investigate how sleep deprivation and sleep depth affect to physiological brain pulsations measured with fast fMRI. Slow vasomotor pulsations were promoted by sleep deprivation during N1 sleep, but no respiratory or cardiac pulsation changes occurred due to sleep deprivation. Brain pulsations were also promoted by sleep depth, including wider spectral and spatial changes in N2 sleep. Respiratory pulsations stabilized in the whole brain and CSF pulsations enhanced only during N2 but not in N1 sleep. We suggest that sleep deprivation and sleep depth, both promote brain pulsations in specific way and thus contribute in changes of brain fluid dynamics.

## Data availability statement

The datasets presented in this article are not readily available because privacy or ethical restrictions. Requests to access the datasets should be directed to HH, heta.helakari@oulu.fi.

## Ethics statement

The studies involving humans were approved by Regional Ethics Committee of the Northern Ostrobothnia Hospital District. The studies were conducted in accordance with the local legislation and institutional requirements. The participants provided their written informed consent to participate in this study.

## Author contributions

HH: Conceptualization, Data curation, Formal analysis, Funding acquisition, Investigation, Methodology, Project administration, Resources, Software, Supervision, Validation, Visualization, Writing – original draft, Writing – review & editing. MJ: Conceptualization, Investigation, Methodology, Validation, Visualization, Writing – original draft, Writing – review & editing. TV: Conceptualization, Formal analysis, Investigation, Methodology, Software, Visualization, Writing – original draft, Writing – review & editing. JT: Conceptualization, Data curation, Formal analysis, Investigation, Methodology, Visualization, Writing – original draft, Writing – review & editing. JP: Conceptualization, Formal analysis, Investigation, Investigation, Methodology, Visualization, Writing – original draft, Writing – review & editing. MK: Conceptualization, Data curation, Formal analysis, Methodology, Visualization, Writing – original draft, Writing – review & editing. SE: Conceptualization, Visualization, Writing – original draft, Writing – review & editing. VP: Conceptualization, Investigation, Visualization, Writing – original draft, Writing – review & editing. JK: Conceptualization, Data curation, Funding acquisition, Investigation, Methodology, Validation, Visualization, Writing – original draft, Writing – review & editing. AE: Conceptualization, Methodology, Visualization, Writing – original draft, Writing – review & editing. NH: Conceptualization, Data curation, Formal analysis, Investigation, Methodology, Resources, Software, Validation, Visualization, Writing – original draft, Writing – review & editing. LR: Conceptualization, Data curation, Formal analysis, Investigation, Methodology, Resources, Validation, Visualization, Writing – original draft, Writing – review & editing. TT: Conceptualization, Investigation, Visualization, Writing – original draft, Writing – review & editing. VKo: Conceptualization, Data curation, Formal analysis, Funding acquisition, Investigation, Methodology, Project administration, Resources, Software, Supervision, Validation, Visualization, Writing – original draft, Writing – review & editing. MN: Conceptualization, Investigation, Supervision, Validation, Visualization, Writing – original draft, Writing – review & editing. VKi: Conceptualization, Data curation, Formal analysis, Funding acquisition, Project administration, Resources, Software, Supervision, Validation, Visualization, Writing – original draft, Writing – review & editing.

## References

[ref1] Allen P. J. Josephs O. Turner R. (2000). A method for removing imaging artifact from continuous EEG recorded during functional MRI. NeuroImage 12, 230–239. doi: 10.1006/nimg.2000.0599, PMID: 10913328

[ref2] Allen P. J. Polizzi G. Krakow K. Fish D. R. Lemieux L. (1998). Identification of EEG events in the MR scanner: the problem of pulse artifact and a method for its subtraction. NeuroImage 8, 229–239. doi: 10.1006/nimg.1998.0361 9758737

[ref1001] American Academy of Sleep Medicine (2017). AASM Scoring Manual. Available at: https://aasm.org/clinical-resources/scoring-manual/

[ref3] Anier A. Lipping T. Ferenets R. Puumala P. Sonkajärvi E. Rätsep I.. (2012). Relationship between approximate entropy and visual inspection of irregularity in the EEG signal, a comparison with spectral entropy. Br. J. Anaesth. 109, 928–934. doi: 10.1093/bja/aes312, PMID: 22936824

[ref4] Assländer J. Zahneisen B. Hugger T. Reisert M. Lee H.-L. LeVan P.. (2013). Single shot whole brain imaging using spherical stack of spirals trajectories. NeuroImage 73, 59–70. doi: 10.1016/j.neuroimage.2013.01.065, PMID: 23384526

[ref5] Birn R. M. Diamond J. B. Smith M. A. Bandettini P. A. (2006). Separating respiratory-variation-related fluctuations from neuronal-activity-related fluctuations in fMRI. NeuroImage 31, 1536–1548. doi: 10.1016/j.neuroimage.2006.02.048, PMID: 16632379

[ref6] Bojarskaite L. Vallet A. Bjørnstad D. M. Gullestad Binder K. M. Cunen C. Heuser K.. (2023). Sleep cycle-dependent vascular dynamics in male mice and the predicted effects on perivascular cerebrospinal fluid flow and solute transport. Nature Commun. 14:36643. doi: 10.1038/s41467-023-36643-5, PMID: 36806170 PMC9941497

[ref7] Borbély A. A. (1982). A two process model of sleep regulation. Hum. Neurobiol. 1, 195–204. PMID: 7185792

[ref8] Borbély A. A. Daan S. Wirz-Justice A. Deboer T. (2016). The two-process model of sleep regulation: a reappraisal. J. Sleep Res. 25, 131–143. doi: 10.1111/jsr.12371, PMID: 26762182

[ref9] Bruzzo A. A. Gesierich B. Santi M. Tassinari C. A. Birbaumer N. Rubboli G. (2008). Permutation entropy to detect vigilance changes and preictal states from scalp EEG in epileptic patients. A preliminary study. Neurol. Sci. 29, 3–9. doi: 10.1007/s10072-008-0851-3, PMID: 18379733

[ref10] Cabiddu R. Cerutti S. Viardot G. Werner S. Bianchi A. M. (2012). Modulation of the sympatho-vagal balance during sleep: frequency domain study of heart rate variability and respiration. Front. Physiol. 3:45. doi: 10.3389/fphys.2012.00045, PMID: 22416233 PMC3299415

[ref11] Chang C. Leopold D. A. Schölvinck M. L. Mandelkow H. Picchioni D. Liu X.. (2016). Tracking brain arousal fluctuations with fMRI. Proc. Natl. Acad. Sci. U. S. A. 113, 4518–4523. doi: 10.1073/pnas.1520613113, PMID: 27051064 PMC4843437

[ref12] Cox R. W. (1996). AFNI: software for analysis and visualization of functional magnetic resonance neuroimages. Comput. Biomed. Res. 29, 162–173. doi: 10.1006/cbmr.1996.0014, PMID: 8812068

[ref13] de Zambotti M. Rosas L. Colrain I. M. Baker F. C. (2017). The sleep of the ring: comparison of the OURA sleep tracker against polysomnography. Behav. Sleep Med. 17, 1–15. doi: 10.1080/15402002.2017.1300587 28323455 PMC6095823

[ref14] Dreha-Kulaczewski S. Joseph A. A. Merboldt K.-D. Ludwig H.-C. Gärtner J. Frahm J. (2015). Inspiration is the major regulator of human CSF flow. J. Neurosci. 35, 2485–2491. doi: 10.1523/JNEUROSCI.3246-14.2015, PMID: 25673843 PMC6605608

[ref15] Duff E. P. Johnston L. A. Xiong J. Fox P. T. Mareels I. Egan G. F. (2008). The power of spectral density analysis for mapping endogenous BOLD signal fluctuations. Hum. Brain Mapp. 29, 778–790. doi: 10.1002/hbm.20601, PMID: 18454458 PMC5441229

[ref16] Duyn J. H. (1997). Steady state effects in fast gradient echo magnetic resonance imaging. Magn. Reson. Med. 37, 559–568. doi: 10.1002/mrm.1910370414 9094078

[ref17] Eide P. K. Pripp A. H. Berge B. Hrubos-Strøm H. Ringstad G. Valnes L. M. (2022). Altered glymphatic enhancement of cerebrospinal fluid tracer in individuals with chronic poor sleep quality. J. Cereb. Blood Flow Metab. 42, 1676–1692. doi: 10.1177/0271678X221090747, PMID: 35350917 PMC9441729

[ref1002] Eide P. K. Vinje V. Pripp A. H. Mardal K.-A. Ringstad G. (2021). Sleep deprivation impairs molecular clearance from the human brain. Brain, 144, 863–874. doi: 10.1093/brain/awab047 33829232

[ref18] Fukunaga M. Horovitz S. G. van Gelderen P. de Zwart J. A. Jansma J. M. Ikonomidou V. N.. (2006). Large-amplitude, spatially correlated fluctuations in BOLD fMRI signals during extended rest and early sleep stages. Magn. Reson. Imaging 24, 979–992. doi: 10.1016/j.mri.2006.04.018, PMID: 16997067

[ref19] Fultz N. E. Bonmassar G. Setsompop K. Stickgold R. A. Rosen B. R. Polimeni J. R.. (2019). Coupled electrophysiological, hemodynamic, and cerebrospinal fluid oscillations in human sleep. Science 366, 628–631. doi: 10.1126/science.aax5440, PMID: 31672896 PMC7309589

[ref20] Gao L. Bai L. Zhang Y. Dai X.-J. Netra R. Min Y.. (2015). Frequency-dependent changes of local resting oscillations in sleep-deprived brain. PLoS One 10:323. doi: 10.1371/journal.pone.0120323, PMID: 25798918 PMC4370559

[ref21] Hablitz L. M. Nedergaard M. (2021). The glymphatic system. Curr. Biol. 31, R1371–R1375. doi: 10.1016/j.cub.2021.08.026 34699796

[ref22] Helakari H. Kananen J. Huotari N. Raitamaa L. Tuovinen T. Borchardt V.. (2019). Spectral entropy indicates electrophysiological and hemodynamic changes in drug-resistant epilepsy – a multimodal MREG study. NeuroImage 22:101763. doi: 10.1016/j.nicl.2019.101763, PMID: 30927607 PMC6444290

[ref23] Helakari H. Korhonen V. Holst S. C. Piispala J. Kallio M. Väyrynen T.. (2022). Human NREM sleep promotes brain-wide vasomotor and respiratory pulsations. J. Neurosci. 42, 2503–2515. doi: 10.1523/JNEUROSCI.0934-21.2022, PMID: 35135852 PMC8944230

[ref24] Hennig J. Kiviniemi V. Riemenschneider B. Barghoorn A. Akin B. Wang F.. (2021). 15 years MR-encephalography. MAGMA 34, 85–108. doi: 10.1007/s10334-020-00891-z, PMID: 33079327 PMC7910380

[ref25] Holstein-Rønsbo S. Gan Y. Giannetto M. J. Rasmussen M. K. Sigurdsson B. Beinlich F. R. M.. (2023). Glymphatic influx and clearance are accelerated by neurovascular coupling. Nat. Neurosci. 26, 1042–1053. doi: 10.1038/s41593-023-01327-2, PMID: 37264158 PMC10500159

[ref26] Horovitz S. G. Fukunaga M. De Zwart J. A. Van Gelderen P. Fulton S. C. Balkin T. J.. (2008). Low frequency BOLD fluctuations during resting wakefulness and light sleep: a simultaneous EEG-fMRI study. Hum. Brain Mapp. 29, 671–682. doi: 10.1002/hbm.20428, PMID: 17598166 PMC6871022

[ref27] Hubbard J. Gent T. C. Hoekstra M. M. B. Emmenegger Y. Mongrain V. Landolt H.-P.. (2020). Rapid fast-delta decay following prolonged wakefulness marks a phase of wake-inertia in NREM sleep. Nat. Commun. 11:16915. doi: 10.1038/s41467-020-16915-0, PMID: 32561733 PMC7305232

[ref28] Hugger T. Zahneisen B. LeVan P. Lee K. J. Lee H.-L. Zaitsev M.. (2011). Fast undersampled functional magnetic resonance imaging using nonlinear regularized parallel image reconstruction. PLoS ONE 6:12. doi: 10.1371/journal.pone.0028822, PMID: 22194921 PMC3237553

[ref29] Huotari N. Raitamaa L. Helakari H. Kananen J. Raatikainen V. Rasila A.. (2019). Sampling rate effects on resting state fMRI metrics. Front. Neurosci. 13:279. doi: 10.3389/fnins.2019.00279, PMID: 31001071 PMC6454039

[ref30] Iliff J. J. Wang M. Zeppenfeld D. M. Venkataraman A. Plog B. A. Liao Y.. (2013). Cerebral arterial pulsation drives paravascular CSF-interstitial fluid exchange in the murine brain. J. Neurosci. 33, 18190–18199. doi: 10.1523/JNEUROSCI.1592-13.2013, PMID: 24227727 PMC3866416

[ref31] Jacobs J. Stich J. Zahneisen B. Assländer J. Ramantani G. Schulze-Bonhage A.. (2014). Fast fMRI provides high statistical power in the analysis of epileptic networks. NeuroImage 88, 282–294. doi: 10.1016/j.neuroimage.2013.10.018, PMID: 24140936

[ref32] Jäntti V. Alahuhta S. Barnard J. Sleigh J. W. (2004). Spectral entropy – what has it to do with anaesthesia, and the EEG? (multiple letters) [3]. Br. J. Anaesth. 93, 150–152. doi: 10.1093/bja/aeh578, PMID: 15192005

[ref33] Järvelä M. Kananen J. Korhonen V. Huotari N. Ansakorpi H. Kiviniemi V. (2022). Increased very low frequency pulsations and decreased cardiorespiratory pulsations suggest altered brain clearance in narcolepsy. Commun. Med. 2:122. doi: 10.1038/s43856-022-00187-4, PMID: 36193214 PMC9525269

[ref34] Jenkinson M. Beckmann C. F. Behrens T. E. J. Woolrich M. W. Smith S. M. (2012). FSL. NeuroImage 62, 782–790. doi: 10.1016/j.neuroimage.2011.09.015 21979382

[ref35] Kananen J. Järvelä M. Korhonen V. Tuovinen T. Huotari N. Raitamaa L.. (2022). Increased interictal synchronicity of respiratory related brain pulsations in epilepsy. J. Cereb. Blood Flow Metab. 42, 1840–1853. doi: 10.1177/0271678X221099703 35570730 PMC9536129

[ref36] Kang J.-E. Lim M. M. Bateman R. J. Lee J. J. Smyth L. P. Cirrito J. R.. (2009). Amyloid-β dynamics are regulated by orexin and the sleep-wake cycle. Science 326, 1005–1007. doi: 10.1126/science.1180962, PMID: 19779148 PMC2789838

[ref37] Kiviniemi V. Wang X. Korhonen V. Keinänen T. Tuovinen T. Autio J.. (2016). Ultra-fast magnetic resonance encephalography of physiological brain activity-Glymphatic pulsation mechanisms? J. Cereb. Blood Flow Metab. 36, 1033–1045. doi: 10.1177/0271678X15622047, PMID: 26690495 PMC4908626

[ref38] Kjaerby C. Andersen M. Hauglund N. Untiet V. Dall C. Sigurdsson B.. (2022). Memory-enhancing properties of sleep depend on the oscillatory amplitude of norepinephrine. Nat. Neurosci. 25, 1059–1070. doi: 10.1038/s41593-022-01102-9, PMID: 35798980 PMC9817483

[ref39] Klose U. Strik C. Kiefer C. Grodd W. (2000). Detection of a relation between respiration and CSF pulsation with an echoplanar technique. J. Magn. Reson. Imaging 11, 438–444. doi: 10.1002/(sici)1522-2586(200004)11:4<438::aid-jmri12>3.0.co;2-o, PMID: 10767073

[ref40] Korhonen V. Hiltunen T. Myllylä T. Wang X. Kantola J. Nikkinen J.. (2014). Synchronous multiscale neuroimaging environment for critically sampled physiological analysis of brain function: hepta-scan concept. Brain Connect. 4, 677–689. doi: 10.1089/brain.2014.0258, PMID: 25131996 PMC4238249

[ref41] Lecci S. Fernandez L. M. J. Weber F. D. Cardis R. Chatton J.-Y. Born J.. (2017). Coordinated infraslow neural and cardiac oscillations mark fragility and offline periods in mammalian sleep. Sci. Adv. 3:e1602026. doi: 10.1126/sciadv.1602026, PMID: 28246641 PMC5298853

[ref42] Lee H.-L. Zahneisen B. Hugger T. LeVan P. Hennig J. (2013). Tracking dynamic resting-state networks at higher frequencies using MR-encephalography. NeuroImage 65, 216–222. doi: 10.1016/j.neuroimage.2012.10.015, PMID: 23069810

[ref43] Liu X. De Zwart J. A. Schölvinck M. L. Chang C. Ye F. Q. Leopold D. A.. (2018). Subcortical evidence for a contribution of arousal to fMRI studies of brain activity. Nat. Commun. 9:395. doi: 10.1038/s41467-017-02815-3, PMID: 29374172 PMC5786066

[ref44] Mahon P. Greene B. R. Lynch E. M. McNamara B. Shorten G. D. (2008). Can state or response entropy be used as a measure of sleep depth? Anaesthesia 63, 1309–1313. doi: 10.1111/j.1365-2044.2008.05675.x 19032298

[ref45] Malik V. Smith D. Lee-Chiong T. Jr. (2012). Respiratory physiology during sleep. Sleep Med. Clin. 7, 497–505. doi: 10.1016/j.jsmc.2012.06.011

[ref46] Marzano C. Ferrara M. Curcio G. De Gennaro L. (2010). The effects of sleep deprivation in humans: topographical electroencephalogram changes in non-rapid eye movement (NREM) sleep versus REM sleep. J. Sleep Res. 19, 260–268. doi: 10.1111/j.1365-2869.2009.00776.x, PMID: 19845849

[ref47] Mehrabadi M. A. Azimi I. Sarhaddi F. Axelin A. Niela-Vilén H. Myllyntausta S.. (2020). Sleep tracking of a commercially available smart ring and smartwatch against medical-grade actigraphy in everyday settings: instrument validation study. JMIR Mhealth Uhealth 8:10. doi: 10.2196/20465 PMC7669442 33038869

[ref48] Mestre H. Tithof J. Du T. Song W. Peng W. Sweeney A. M.. (2018). Flow of cerebrospinal fluid is driven by arterial pulsations and is reduced in hypertension. Nat. Commun. 9:4878. doi: 10.1038/s41467-018-07318-3, PMID: 30451853 PMC6242982

[ref1003] Moehlman T. M. de Zwart J. A. Chappel-Farley M. G. Liu X. McClain I. B. Chang C. (2019). All-night functional magnetic resonance imaging sleep studies. J. Neurosci. Methods, 316, 83–98. doi: 10.1016/j.jneumeth.2018.09.019 30243817 PMC6524535

[ref49] Özbay P. S. Chang C. Picchioni D. Mandelkow H. Chappel-Farley M. G. van Gelderen P.. (2019). Sympathetic activity contributes to the fMRI signal. Commun. Biol. 2:1. doi: 10.1038/s42003-019-0659-0 31754651 PMC6861267

[ref50] Picchioni D. Özbay P. S. Mandelkow H. de Zwart J. A. Wang Y. van Gelderen P.. (2022). Autonomic arousals contribute to brain fluid pulsations during sleep. NeuroImage 249:118888. doi: 10.1016/j.neuroimage.2022.118888 35017126 PMC11395500

[ref51] Posse S. Ackley E. Mutihac R. Zhang T. Hummatov R. Akhtari M.. (2013). High-speed real-time resting-state fMRI using multi-slab echo-volumar imaging. Front. Hum. Neurosci. 7:479. doi: 10.3389/fnhum.2013.00479, PMID: 23986677 PMC3752525

[ref52] Raitamaa L. Huotari N. Korhonen V. Helakari H. Koivula A. Kananen J.. (2021). Spectral analysis of physiological brain pulsations affecting the BOLD signal. Hum. Brain Mapp. 42, 4298–4313. doi: 10.1002/hbm.25547, PMID: 34037278 PMC8356994

[ref53] Rajna Z. Mattila H. Huotari N. Tuovinen T. Krüger J. Holst S. C.. (2021). Cardiovascular brain impulses in Alzheimer’s disease. Brain 144, 2214–2226. doi: 10.1093/brain/awab144, PMID: 33787890 PMC8422353

[ref54] Rajna Z. Raitamaa L. Tuovinen T. Heikkila J. Kiviniemi V. Seppanen T. (2019). 3D multi-resolution optical flow analysis of cardiovascular pulse propagation in human brain. IEEE Trans. Med. Imaging 38, 2028–2036. doi: 10.1109/TMI.2019.2904762, PMID: 30892202

[ref55] Rasmussen M. K. Mestre H. Nedergaard M. (2018). The glymphatic pathway in neurological disorders. Lancet Neurol. 17, 1016–1024. doi: 10.1016/S1474-4422(18)30318-1, PMID: 30353860 PMC6261373

[ref56] Sattin D. Duran D. Visintini S. Schiaffi E. Panzica F. Carozzi C.. (2021). Analyzing the loss and the recovery of consciousness: functional connectivity patterns and changes in heart rate variability during Propofol-induced anesthesia. Front. Syst. Neurosci. 15:2080. doi: 10.3389/fnsys.2021.652080 PMC8055941 33889078

[ref57] Shannon C. E. (1948). The mathematical theory of communication. Bell Syst. Tech. J. 27, 623–656. doi: 10.1002/j.1538-7305.1948.tb00917.x

[ref58] Shokri-Kojori E. Wang G.-J. Wiers C. E. Demiral S. B. Guo M. Kim S. W.. (2018). ß-amyloid accumulation in the human brain after one night of sleep deprivation. Proc. Natl. Acad. Sci. U. S. A. 115, 4483–4488. doi: 10.1073/pnas.1721694115, PMID: 29632177 PMC5924922

[ref59] Sloots J. J. Biessels G. J. Zwanenburg J. J. M. (2020). Cardiac and respiration-induced brain deformations in humans quantified with high-field MRI. NeuroImage 210:116581. doi: 10.1016/j.neuroimage.2020.116581, PMID: 31982580

[ref60] Smith S. M. (2002). Fast robust automated brain extraction. Hum. Brain Mapp. 17, 143–155. doi: 10.1002/hbm.10062, PMID: 12391568 PMC6871816

[ref61] Somers V. K. Dyken M. E. Mark A. L. Abboud F. M. (1993). Sympathetic-nerve activity during sleep in Normal subjects. N. Engl. J. Med. 328, 303–307. doi: 10.1056/NEJM199302043280502 8419815

[ref62] Song C. Boly M. Tagliazucchi E. Laufs H. Tononi G. (2022). fMRI spectral signatures of sleep. Proc. Natl. Acad. Sci. U. S. A. 119:e2016732119. doi: 10.1073/pnas.2016732119, PMID: 35862450 PMC9335231

[ref63] Sowho M. Amatoury J. Kirkness J. P. Patil S. P. (2014). Sleep and respiratory physiology in adults. Clin. Chest Med. 35, 469–481. doi: 10.1016/j.ccm.2014.06.002 25156763

[ref64] Tabuchi M. Lone S. R. Liu S. Liu Q. Zhang J. Spira A. P.. (2015). Sleep interacts with aβ to modulate intrinsic neuronal excitability. Curr. Biol. 25, 702–712. doi: 10.1016/j.cub.2015.01.016, PMID: 25754641 PMC4366315

[ref65] Tagliazucchi E. Laufs H. (2014). Decoding wakefulness levels from typical fMRI resting-state data reveals reliable drifts between wakefulness and sleep. Neuron 82, 695–708. doi: 10.1016/j.neuron.2014.03.020, PMID: 24811386

[ref66] Tuovinen T. Kananen J. Rajna Z. Lieslehto J. Korhonen V. Rytty R.. (2020). The variability of functional MRI brain signal increases in Alzheimer’s disease at cardiorespiratory frequencies. Sci. Rep. 10:1. doi: 10.1038/s41598-020-77984-1, PMID: 33298996 PMC7726142

[ref67] Urigüen J. A. García-Zapirain B. Artieda J. Iriarte J. Valencia M. (2017). Comparison of background EEG activity of different groups of patients with idiopathic epilepsy using Shannon spectral entropy and cluster-based permutation statistical testing. PLoS One 12:e0184044. doi: 10.1371/journal.pone.0184044, PMID: 28922360 PMC5602520

[ref68] Vakkuri A. Yli-Hankala A. Sandin R. Mustola S. Høymork S. Nyblom S.. (2005). Spectral entropy monitoring is associated with reduced propofol use and faster emergence in propofol-nitrous oxide-alfentanil anesthesia. Anesthesiology 103, 274–279. doi: 10.1097/00000542-200508000-00010, PMID: 16052109

[ref69] van Veluw S. J. Hou S. S. Calvo-Rodriguez M. Arbel-Ornath M. Snyder A. C. Frosch M. P.. (2020). Vasomotion as a driving force for Paravascular clearance in the awake mouse brain. Neuron 105, 549–561.e5. doi: 10.1016/j.neuron.2019.10.033, PMID: 31810839 PMC7028316

[ref70] Viertiö-Oja H. Maja V. Särkelä M. Talja P. Tenkanen N. Tolvanen-Laakso H.. (2004). Description of the entropy™ algorithm as applied in the Datex-Ohmeda 5/5™ entropy module. Acta Anaesthesiol. Scand. 48, 154–161. doi: 10.1111/j.0001-5172.2004.00322.x, PMID: 14995936

[ref71] Vinje V. Ringstad G. Lindstrøm E. K. Valnes L. M. Rognes M. E. Eide P. K.. (2019). Respiratory influence on cerebrospinal fluid flow – a computational study based on long-term intracranial pressure measurements. Sci. Rep. 9:9732. doi: 10.1038/s41598-019-46055-5, PMID: 31278278 PMC6611841

[ref72] Von Schulthess G. K. Higgins C. B. (1985). Blood flow imaging with MR: spin-phase phenomena. Radiology 157, 687–695. doi: 10.1148/radiology.157.3.2997836, PMID: 2997836

[ref73] Winer J. R. Mander B. A. Kumar S. Reed M. Baker S. L. Jagust W. J.. (2020). Sleep disturbance forecasts β-amyloid accumulation across subsequent years. Curr. Biol. 30, 4291–4298.e3. doi: 10.1016/j.cub.2020.08.017, PMID: 32888482 PMC7642104

[ref74] Wise R. G. Ide K. Poulin M. J. Tracey I. (2004). Resting fluctuations in arterial carbon dioxide induce significant low frequency variations in BOLD signal. NeuroImage 21, 1652–1664. doi: 10.1016/j.neuroimage.2003.11.025, PMID: 15050588

[ref75] Xie L. Kang H. Xu Q. Chen M. J. Liao Y. Thiyagarajan M.. (2013). Sleep drives metabolite clearance from the adult brain. Science 342, 373–377. doi: 10.1126/science.1241224, PMID: 24136970 PMC3880190

[ref76] Yamada S. Miyazaki M. Yamashita Y. Ouyang C. Yui M. Nakahashi M.. (2013). Influence of respiration on cerebrospinal fluid movement using magnetic resonance spin labeling. Fluids Barriers CNS 10:1. doi: 10.1186/2045-8118-10-36, PMID: 24373186 PMC3895787

[ref77] Yildiz S. Grinstead J. Hildebrand A. Oshinski J. Rooney W. D. Lim M. M.. (2022). Immediate impact of yogic breathing on pulsatile cerebrospinal fluid dynamics. Sci. Rep. 12:10894. doi: 10.1038/s41598-022-15034-8, PMID: 35764793 PMC9240010

[ref78] Yokoba M. Hawes H. G. Kieser T. M. Katagiri M. Easton P. A. (2016). Parasternal intercostal and diaphragm function during sleep. J. Appl. Physiol. 121, 59–65. doi: 10.1152/japplphysiol.00508.2015, PMID: 27125847

[ref79] Zahneisen B. Hugger T. Lee K. J. Levan P. Reisert M. Lee H.-L.. (2012). Single shot concentric shells trajectories for ultra fast fMRI. Magn. Reson. Med. 68, 484–494. doi: 10.1002/mrm.23256, PMID: 22131236

