# Supplementary material for: Effect of sleep deprivation and NREM sleep stage on physiological brain pulsations
Source: Front Neurosci. 2023 Dec 1;17:1275184. doi: 10.3389/fnins.2023.1275184 (PMC10722275; doi:10.3389/fnins.2023.1275184)
Supplement: Supplementary file 1 [file Table_1.docx]

**Supplementary table 1. 23 subjects that were included in the analysis (ID1-13 sleep deprived, SD and ID14-23 non-sleep deprived, NSD).** ID13 data was taken from eyes closed N1 stage scanned in the afternoon, and were therefore not included in SD vs. NSD analysis. Awake data was available from all subjects, N1 data from 21 subjects and N2 data from 14 subjects. SD vs. NSD comparison included 12 SD subjects and 8 NSD subjects.

| Subject ID | Awake | N1 | N2 | SD vs. NSD |
| --- | --- | --- | --- | --- |
| 1 | x | x | x | x |
| 2 | x | x | - | x |
| 3 | x | x | x | x |
| 4 | x | x | x | x |
| 5 | x | x | x | x |
| 6 | x | x | x | x |
| 7 | x | x | x | x |
| 8 | x | x | x | x |
| 9 | x | x | x | x |
| 10 | x | x | - | x |
| 11 | x | x | x | x |
| 12 | x | x | x | x |
| 13 | x | x | - | - |
| 14 | x | x | - | x |
| 15 | x | - | x | - |
| 16 | x | x | - | x |
| 17 | x | - | x | - |
| 18 | x | x | - | x |
| 19 | x | x | - | x |
| 20 | x | x | - | x |
| 21 | x | x | x | x |
| 22 | x | x | x | x |
| 23 | x | x | - | x |
