# Supplementary material for: Effect of sleep deprivation and NREM sleep stage on physiological brain pulsations
Source: Front Neurosci. 2023 Dec 1;17:1275184. doi: 10.3389/fnins.2023.1275184 (PMC10722275; doi:10.3389/fnins.2023.1275184)
Supplement: Supplementary file 2 [file Table_2.docx]

**Supplementary table 2. Smart ring data characteristics from the subjects in data set 1.** Table presents the duration of sleep deprivation (h) before Sleep scan session and amount of sleep (min) before Awake scan session and before Sleep scan session.

|  | Duration | Sleep before | Sleep before |
| --- | --- | --- | --- |
| Subject ID | of SD (h) | Awake scan (min) | Sleep scan (min) |
|  |  |  |  |
| 1 | 21,1 | 543 | 0 |
| 2 | 26,2 | 433 | 73 |
| 3 | 25,3 | 366 | 0 |
| 4 | 24,5 | 618 | 24 |
| 5 | - | 362 | - |
| 6 | 22,8 | 444 | 5 |
| 7 | 23,9 | 554 | 9 |
| 8 | 24,8 | 406 | 0 |
| 9 | 24,3 | 478 | 0 |
| 10 | 24 | 354 | 0 |
| 11 | 24,1 | 529 | 13 |
| 12 | 24,4 | 445 | 0 |
| 13 | 26,4 | 478 | 2 |
